# Waves of sumoylation support transcription dynamics during adipocyte differentiation

**DOI:** 10.1093/nar/gkac027

**Published:** 2022-01-31

**Authors:** Xu Zhao, Ivo A Hendriks, Stéphanie Le Gras, Tao Ye, Lucía Ramos-Alonso, Aurélie Nguéa P, Guro Flor Lien, Fatemeh Ghasemi, Arne Klungland, Bernard Jost, Jorrit M Enserink, Michael L Nielsen, Pierre Chymkowitch

**Affiliations:** Department of Biosciences, Faculty of Mathematics and Natural Sciences, University of Oslo, 0316 Oslo, Norway; Department of Microbiology, Oslo University Hospital, 0372 Oslo, Norway; Proteomics Program, Novo Nordisk Foundation Center for Protein Research (NNF-CPR), Faculty of Health and Medical Sciences, University of Copenhagen, 2200 Copenhagen, Denmark; Institut de Génétique et de Biologie Moléculaire et Cellulaire, CNRS UMR7104, Inserm U964, Université de Strasbourg, Illkirch, France; Institut de Génétique et de Biologie Moléculaire et Cellulaire, CNRS UMR7104, Inserm U964, Université de Strasbourg, Illkirch, France; Department of Biosciences, Faculty of Mathematics and Natural Sciences, University of Oslo, 0316 Oslo, Norway; Department of Microbiology, Oslo University Hospital, 0372 Oslo, Norway; Department of Microbiology, Oslo University Hospital, 0372 Oslo, Norway; Department of Microbiology, Oslo University Hospital, 0372 Oslo, Norway; Department of Biosciences, Faculty of Mathematics and Natural Sciences, University of Oslo, 0316 Oslo, Norway; Department of Biosciences, Faculty of Mathematics and Natural Sciences, University of Oslo, 0316 Oslo, Norway; Department of Microbiology, Oslo University Hospital, 0372 Oslo, Norway; Institut de Génétique et de Biologie Moléculaire et Cellulaire, CNRS UMR7104, Inserm U964, Université de Strasbourg, Illkirch, France; Department of Biosciences, Faculty of Mathematics and Natural Sciences, University of Oslo, 0316 Oslo, Norway; Department of Molecular Cell Biology, Institute for Cancer Research, Oslo University Hospital, 0372 Oslo, Norway; Centre for Cancer Cell Reprogramming, Institute of Clinical Medicine, Faculty of Medicine, University of Oslo, 0318 Oslo, Norway; Proteomics Program, Novo Nordisk Foundation Center for Protein Research (NNF-CPR), Faculty of Health and Medical Sciences, University of Copenhagen, 2200 Copenhagen, Denmark; Department of Biosciences, Faculty of Mathematics and Natural Sciences, University of Oslo, 0316 Oslo, Norway; Department of Microbiology, Oslo University Hospital, 0372 Oslo, Norway

## Abstract

Tight control of gene expression networks required for adipose tissue formation and plasticity is essential for adaptation to energy needs and environmental cues. However, the mechanisms that orchestrate the global and dramatic transcriptional changes leading to adipocyte differentiation remain to be fully unraveled. We investigated the regulation of nascent transcription by the sumoylation pathway during adipocyte differentiation using SLAMseq and ChIPseq. We discovered that the sumoylation pathway has a dual function in differentiation; it supports the initial downregulation of pre-adipocyte-specific genes, while it promotes the establishment of the mature adipocyte transcriptional program. By characterizing endogenous sumoylome dynamics in differentiating adipocytes by mass spectrometry, we found that sumoylation of specific transcription factors like PPARγ/RXR and their co-factors are associated with the transcription of adipogenic genes. Finally, using RXR as a model, we found that sumoylation may regulate adipogenic transcription by supporting the chromatin occurrence of transcription factors. Our data demonstrate that the sumoylation pathway supports the rewiring of transcriptional networks required for formation of functional adipocytes. This study also provides the scientists in the field of cellular differentiation and development with an in-depth resource of the dynamics of the SUMO-chromatin landscape, SUMO-regulated transcription and endogenous sumoylation sites during adipocyte differentiation.

## Graphical Abstract

**Graphical Abstract:**
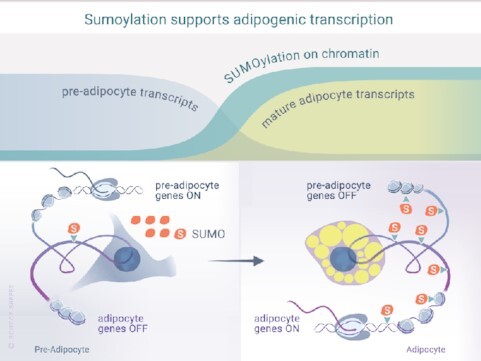
Waves of sumoylation support transcription dynamics during adipocyte differentiation.

## INTRODUCTION

Post-translational modification by the small ubiquitin-like modifier SUMO is a conserved, essential and versatile process playing a critical yet poorly understood role in cell differentiation, identity, growth and adaptation to various stimuli (1–3). Mammals express three major SUMO isoforms: SUMO-1, -2 and -3. SUMO-2 and -3 are nearly identical and are usually referred to as SUMO-2/3. SUMO precursors are matured by SUMO-specific proteases (SENPs), after which they are linked to the E1 activating enzyme SAE1/SAE2. SUMO is then transferred to the E2 conjugating enzyme UBC9, which conjugates SUMO to lysine residues of target proteins in a process that is often facilitated by E3 ligases. Sumoylation can alter the stability, conformation, interactions or subcellular localization of target proteins. SUMO targets can be desumoylated by SENPs (2).

Proteomic studies have shown that most SUMO targets are involved in chromatin regulation, chromosome integrity, mRNA processing and transcription (4–6). Consistently, genome-wide chromatin immunoprecipitation sequencing (ChIPseq) experiments revealed that SUMO is present at gene promoters, intragenic regions, enhancers and transcription factor binding sites (7–11). The SUMO chromatin landscape is substantially remodeled in response to heat shock, inflammation, oncogene-induced senescence, nutrient deprivation and pro-growth signals (7,8,10,12–14).

Recent studies have revealed that SUMO is also important for cellular identity (9,15). However, these studies only compared undifferentiated versus terminally differentiated cells, precluding the sumoylation dynamics that may occur throughout the differentiation process. Therefore, there exists a need for a biologically relevant model to study sumoylome dynamics during differentiation.

Interestingly, mouse knockout studies have revealed that animals lacking *Sumo-1* or *Senp2* are resistant to high fat diet (HFD)-induced obesity (16,17), whereas specific loss of *Ubc9* in white adipose tissue triggers lipoatrophy (18). Consistently, loss of *Sumo-1, Ubc9, Senp1 or Senp2* in cellular models impedes adipogenesis and is associated with altered expression of genes under the control of PPARγ, cEBPα or cEBPδ, as well as genes involved in lipid and energy metabolism (19–21). This suggests that formation of the adipose tissue depends on the sumoylation pathway, although the dynamics of this process remain very poorly described.

In this study, we used adipocyte differentiation (AD) as a model system to uncover the dynamics of the sumoylome, the SUMO-chromatin landscape and SUMO-dependent gene transcription during differentiation. Our data show that SUMO plays a dual role during adipogenesis by supporting the transcription of pre-adipocyte genes and supporting adipogenic genes transcription to establish adipocyte identity.

## MATERIALS AND METHODS

### 3T3-L1 culture, differentiation and treatments

3T3-L1 preadipocytes (CL-173, ATCC) were maintained in Dulbecco’s modified Eagle’s medium (DMEM) supplemented with 10% bovin calf serum (12138C, Sigma) and 1% Penicillin/Streptavidin (P/S) at 37°C in a humidified incubator in a 5% CO_2_ in air atmosphere.

Before adipogenic induction 3T3-L1 preadipocytes were grown to confluence by replacing the maintenance medium every second day for at least 4 days. Differentiation of 3T3-L1 cells was induced with differentiation medium (DMEM supplemented with 10% fetal bovine serum (F7524, Sigma), 1 mM dexamethasone (D4902, Sigma), 0.5 mM 3-isobutyl-1-methylxanthine (I5879, Sigma), 10 μg/ml insulin and 1% P/S). At day 3 post adipogenic induction, the differentiation medium was replaced with the adipocyte maintenance medium (DMEM supplemented with 10% FBS, 10 μg/ml of insulin and 1% P/S). Adipocyte maintenance medium was then refreshed every second day. SUMOylation was inhibited by supplementing the culture medium with 0.5 μM of ML-792 (407886, Medkoo Biosite).

### Cell viability assay

3T3-L1 cells were grown and induced to differentiation as described in *‘3T3-L1 culture, differentiation and treatment’* in a tissue culture 96-well plate with clear bottom. Cells were treated with DMSO, 0.5 μM or 1 μM of ML-792 from Day −2. The cell viability at each individual time point was assessed with Cell Counting Kit 8 (Abcam, ab228554). Briefly, 10 μl of CCK8 solution was added into 100 μl medium as instructed. Cells were incubated 37°C from dark for 1 h. After incubation, the absorbance at 460 nm was read by the microplate reader (PerkinElmer, VICTOR Nivo). The absorbance of the blank wells with only medium containing CCK8 was subtracted from the values for those wells with cells. At least 5 parallel wells were used per treatment and per time point.

### Lipid droplet staining

3T3-L1 cells were grown as described in ‘3T3-L1 culture, differentiation and treatment’. At day 7 post induction cells were incubated in a medium containing 50 μM of Mitotracker (Cell Signaling, 9082P) for 30 min. Cells were then washed with PBS and fixed with 4% formaldehyde during 10 min and rinsed 3 times with PBS. Lipid droplets were stained with a Bodipy 493/503 (D3922, Thermo Fisher Scientific) in a staining solution (1 μg/ml Bodipy 493/503, 150 mM NaCl) for 10 min at room temperature. Nuclei were stained with DAPI (0.5 μg/ml).

### Image acquisition

Images were acquired using a Leica confocal SP8 device. Widefield images were acquired using the 40 X S Plan Fluor ELWD objective as Z-series (step sizes 0.2–1 μm, depending on the staining). At least 3 wells and 8 representative fields per well were captured.

### Image analysis and quantification

All images were analyzed using the FIJI software (Fiji is just ImageJ) (National Institutes of Health, Bethesda, MD, USA). Z series images were corrected from bleaching then projected to obtain a ‘focused’ single image corresponding to the different focal planes, using the *Stack Focuser* plugin (https://imagiej.nih.gov/ij/plugins/stack-focuser.html). Mitotracker images were used to delineate cell shape and assign lipid droplets to corresponding cells. Lipid droplets were segmented and quantified in single cell using CellpProfiler v4.2.1 (22).

### QuantSeq and SLAM-Sequencing sample preparation

3T3-L1 cells were grown as described in ‘*Adipocyte culture, differentiation and treatment*’ and treated with DMSO or ML-792. Three biological replicates were harvested per condition. Prior harvesting, nascent RNAs were labeled with 100 μM of 4-thiouridine during 45 min. After labeling cells were lysed, homogenized and collected using 1 ml of RNAzol®RT (RN190-100, Molecular Research Center) before adding 0.4 ml of water per 1 ml of homogenate. The mixture was vortexed for 5 min and centrifuged at 12 000 *g* for 15 min. Following centrifugation, DNA, proteins and polysaccharides form a semisolid pellet at the bottom of the tube. The RNA remains soluble in the supernatant. The supernatant (1 ml maximum) was transferred to a new tube and 1 volume of 70% ethanol was added. The mixture was homogenized by pipetting (do not centrifuge). Samples were then applied to a RNeasy spin column (74104, Qiagen), and RNAs were purified according to Qiagen’s instructions. Base conversion was performed using the SLAMseq catabolic kinetics module (Lexogen, 062.24). RNA quantification and quality control were performed using Tape Station 4150 (Agilent).

### Library preparation and sequencing of QuantSeq and SLAMseq samples

mRNA-Seq libraries were generated according to manufacturer’s instructions from 500 ng of total RNA using the QuantSeq 3′mRNA‐Seq Library Prep Kit for Illumina (FWD) (# 015, Lexogen GmbH, Vienna, Austria). Reverse transcription was initiated by oligo dT priming. After first strand cDNA synthesis the RNA was removed and second strand synthesis was initiated by random priming. Oligo dT primer and random primers contain Illumina-compatible adapter sequences. The resulting double-stranded cDNA was then purified and PCR amplified (30 s at 98°C; [10 s at 98°C, 20 s at 65°C, 30 s at 72°C] × 11 cycles; 1 min at 72°C), introducing i7 indexes. Surplus PCR primers were further removed by purification using SPRI-select beads (Beckman-Coulter, Villepinte, France), and the final libraries were checked for quality and quantified using capillary electrophoresis. The libraries were sequenced on Illumina Hiseq 4000 sequencer as Single-Read 50 base reads following Illumina’s instructions. Image analysis and base calling were performed using RTA 2.7.7 and bcl2fastq. 2.17.1.14. Adapter dimer reads were removed using DimerRemover (https://sourceforge.net/projects/dimerremover/).

### SLAMseq and QuantSeq data analysis

mm10 mouse genome assembly and Refseq 3′UTR coordinates were downloaded from UCSC (4 August 2020) using Table Browser. Sequencing reads were mapped and filtered with SlamDunk pipeline v0.4.3 (http://t-neumann.github.io/slamdunk/docs.html #document-Dunks). SlamDunk all (http://t-neumann.github.io/slamdunk/docs.html#all) was applied to full analysis for all samples. Reads with ≥ 1 T > C conversions were considered as labeled reads. Default settings for other parameters was followed.

Two parallel differential gene expression analyses were performed using DESeq2 R package (1.26.0). The total RNA reads were used for Quantseq analysis. The normalization size factor in Quantseq analysis was applied to global normalization for labeled reads. Time course experiments design (http://master.bioconductor.org/packages/release/workflows/vignettes/rnaseqGene/inst/d

oc/rnaseqGene.html#time-course-experiments) was adapted for our data. Principal component analysis was performed after variance stabilizing transformation on total genes for both analyses.

### Chromatin immunoprecipitation (ChIP)

3T3-L1 cells were grown as described in ‘*Adipocyte culture, differentiation and treatment*’ in 15 cm dishes. Two dishes were used per biological replicate and two biological replicates were collected for each time point.

Our ChIP procedure was adapted from (23). Cells were crosslinked in dish with 1% formaldehyde for 8 min. Formaldehyde was then neutralized using 125 mM glycine for 10 min. After two washes with cold PBS, the cells were collected in the Lysis buffer (5 mM PIPES pH 7.5, 85 mM KCl, 0.5% NP-40, 20 mM N-ethyl maleimide [NEM] and protease inhibitor cocktail [04693159001, Roche]) and incubated at 4°C for 10 min with rotation. Nuclei were centrifuged (1500 rpm for 10 min at 4°C) and resuspended in a nucleus lysis buffer (50 mM Tris-HCl pH 7.5, 1% SDS, 10 mM EDTA, 20 mM NEM and protease inhibitor cocktail) and incubated at 4°C for 2 h. Lysates were sonicated for 15 cycles (30 s on/30 s off) at 4°C using a Bioruptor Pico sonicator (Diagenode). After sonication, lysates were centrifuged at 14 000 rpm for 10 min at 4°C. Protein concentration was assessed using the Bradford assay and 250 μg of chromatin were used for each immunoprecipitation. Input samples (12.5 μg) were saved. Samples were diluted 10-fold in the immunoprecipitation buffer (1.1% Triton X100, 50 mM Tris-HCl, pH 7.5, 167 mM NaCl, 5 mM N-ethyl maleimide, 1 mM EDTA, 0.01% SDS and protease inhibitor cocktail). Immunoprecipitations were carried out a SUMO-2/3 antibody (ab3742, Abcam) or a RXR antibody (433900, Thermo Fisher Scientific) and 420 μl of Dynabeads Protein A or G (Thermo Fisher Scientific) overnight at 4°C. Beads were then washed 2 times in low-salt buffer (50 mM Tris-HCl, pH 7.5, 150 mM NaCl, 1% Triton X100, 0.1% SDS, 1 mM EDTA), 2 times in high-salt buffer (50 mM Tris-HCl, pH 7.5, 500 mM NaCl, 1% Triton X100, 0.1% SDS, 1 mM EDTA), 2 times in LiCl buffer (20 mM Tris-HCl, pH 7.5, 250 mM LiCl, 1% NP-40, 1% deoxycholic acid, 1 mM EDTA) and in TE buffer (10 mM Tris-HCl, pH 7.5, 0.2% Tween20, 1 mM EDTA). Elution was done two time in 50 μl of 100 mM NaHCO_3_, 1% SDS at 65°C for 10 min under agitation. Chromatin crosslinking was reversed at 65°C for 5 h with 280 mM NaCl and 0.2 μg/ml RNase DNase free (11119915001, Roche). Proteins were then digested using 0.2 μg/ml of Proteinase K (03115828001, Roche) during 1 h at 65°C. DNA from immunoprecipitations and inputs were purified using the Qiagen PCR purification kit. DNA concentration was assessed using a Qubit device (Q32866, Invitrogen).

### Library preparation and sequencing of ChIP samples (ChIPseq)

ChIP samples were purified using Agencourt AMPure XP beads (Beckman Coulter) and quantified with the Qubit (Invitrogen). ChIPseq libraries were prepared from 10 ng of double-stranded purified DNA using the MicroPlex Library Preparation kit v2 (C05010014, Diagenode s.a., Seraing, Belgium), according to manufacturer’s instructions. In the first step, the DNA was repaired and yielded molecules with blunt ends. In the next step, stem-loop adaptors with blocked 5 prime ends were ligated to the 5 prime end of the genomic DNA, leaving a nick at the 3 prime end. The adaptors cannot ligate to each other and do not have single-strand tails, avoiding non-specific background. In the final step, the 3 prime ends of the genomic DNA were extended to complete library synthesis and Illumina compatible indexes were added through a PCR amplification (7 cycles). Amplified libraries were purified and size-selected using Agencourt AMPure XP beads (Beckman Coulter) to remove unincorporated primers and other reagents. The libraries were sequenced on Illumina Hiseq 4000 sequencer as Single-Read 50 base reads following Illumina's instructions. Image analysis and base calling were performed using RTA 2.7.7 and bcl2fastq 2.17.1.14. Adapter dimer reads were removed using DimerRemover.

### ChIPseq sequencing data analysis

Reads were mapped to the Mus musculus genome (assembly mm10) using Bowtie (24) v1.0.0 using the following parameters -m 1 –strata –best. Reads mapped in genomic regions flagged as ENCODE blacklist were removed (25). SUMO peaks were called with the ENCODE ChIPseq pipeline v1.3.6. Briefly, the pipeline ran quality controls and called peaks with spp v1.15.5 (26). Reproductible peaks were kept after the IDR analysis was run (optimal IDR sets of peaks were kept). Peaks were annotated relative to genomic features using Homer annotatePeaks.pl v4.11.1 (27). Known or *de novo* TF motifs were identified using HOMER findMotifsGenome.pl with default parameters. Heatmaps and mean profiles presenting read enrichments at various genomic locations were generated using Easeq software v1.111 (28). To compare SUMO peaks enrichments over time, the union of all peak positions was computed with BEDtools v2.26.0 (29). Then, read counts per peak (union peak set) were normalized across libraries with the method proposed by Anders and Huber (30) and implemented in the Bioconductor package v1.24.0 (31). Regions varying due to time effect were identified using a likelihood ratio test (LRT) with DESeq2. Resulting *P*-values were adjusted for multiple testing using the Benjamin and Hochberg method (32). Significant regions were those which adjusted *P*-value $ \le$ 0.05, absolute fold change $ >$ 1.5.

### Analysis of previous RXR and PPARγ ChIPseq datasets

RXR and PPARγ data were mapped to the Mus musculus genome (assembly mm10) using Bowtie (24) v1.0.0 using the following parameters -m 1 –strata –best. Peaks were called using MACS2 callpeak with default parameters except for -g mm –nomodel –extsize 200. Peaks were annotated relative to genomic features using Homer annotatePeaks.pl v4.11.1 (27). For integrating SUMO-2/3, RXR and PPARγ ChIPseq, heatmaps were generated using Deeptools v3.5 using the tool bamCoverage to generate bigwigs files with a step of 10 nt. Bigwig files were normalized using the CPM (counts per million mapped reads) method (33). Then, the tool Deeptools computeMatrix v3.5 was used to generate a count matrix at the positions of interest and finally the tool Deeptools plotProfile v3.5 were used to generate mean profile plots.

### SUMO mass spectrometry

3T3-L1 cells were grown as described in ‘*Adipocyte culture, differentiation and treatment*’. Four biological replicates were collected for each condition. Cells were washed with PBS supplemented with 20 mM NEM (E3876, Merck Life Science). Cells were vigorously lysed in guanidium lysis buffer (6 M guanidine-HCl, 50 mM Tris pH 8.5, 20 mM NEM), after which they were immediately snap frozen. Lysates were stored at −80°C until further processing. In essence, sample preparation and SUMO-IP for native and endogenous mass spectrometry (MS) analysis was performed as described previously (4). Lysates were thawed at room temperature, after which they were supplemented with 5 mM chloroacetamide (CAA) and 5 mM Tris(2-carboxyethyl)phosphine (TCEP). Samples were homogenized via sonication using a microtip sonicator, at 30 W using three 10 s pulses, and afterwards cleared by centrifugation at 4250 *g*. Endoproteinase Lys-C (Wako) was added to samples in a 1:200 enzyme-to-protein ratio (w/w). Digestion was performed overnight, still, and at room temperature. Digested samples were diluted with three volumes of 50 mM ammonium bicarbonate (ABC), and a second round of overnight digestion was performed by addition of Lys-C in a 1:200 enzyme-to-protein ratio. Digests were acidified by addition of 0.5% trifluoroacetic acid (TFA), 1:100 vol/vol from a 50% TFA stock, after which they were transferred to 50 ml tubes and centrifuged at 4250 *g* and at 4°C for 30 min. Clarified digests were carefully decanted into clean 50 ml tubes, after which peptides were purified using C8 Sep-Pak cartridges (Waters) according to the manufacturer’s instructions. Sep-Pak cartridges with 500 mg C8 sorbent were used, with one cartridge used for each ∼25 mg of digested protein. Small and hydrophilic peptides were pre-eluted using 5 ml of 20% acetonitrile (ACN) in 0.1% TFA, and 3 ml of 25% ACN in 0.1% TFA. SUMOylated peptides were eluted using 1 ml of 35% ACN in 0.1 TFA, 1 ml of 40% ACN in 0.1% TFA and 2 ml of 45% ACN in 0.1% TFA. For each replicate sample, all SepPak elutions were pooled in 50 ml tubes with small holes punctured into the caps, and then frozen overnight at −80°C. Deep-frozen samples were lyophilized to dryness for 48 h, with the pressure target set at 0.004 mbar and the condenser coil at −85°C.

### Crosslinking of SUMO antibody to beads

Overall, 750 μl of Protein G Agarose beads (Roche) were used to capture 400 μl of SUMO-2/3 antibody (8A2, acquired from Abcam, ab81371; ∼5–10 μg/μl antibody). All washing and handling steps were followed by centrifugation of the beads at 500 *g* for 3 min in a swing-out centrifuge with delayed deceleration and careful aspiration of buffers, to minimize loss of beads. Beads were pre-washed 4 times with ice-cold PBS, split across three 1.5 ml tubes, after which the antibody was added and the tubes filled completely with ice-cold PBS. Beads and antibody were incubated at 4°C on a rotating mixer for 1 h and subsequently washed 3 times with ice-cold PBS. Crosslinking of the antibody to the beads was achieved by addition of 1 ml of 0.2 M sodium borate, pH 9.0, which was freshly supplemented with 20 mM dimethyl pimelimidate (DMP). Crosslinking was performed for 30 min at room temperature on a rotating mixer, after which the crosslinking step was repeated once. SUMO-IP beads where then washed twice with ice-cold PBS, twice with 0.1 M glycine pH 2.8, and three times with ice-cold PBS, after which all beads were pooled in a single 1.5 ml tube and stored until use at 4°C in PBS supplemented with 10 mM sodium azide.

### Purification of SUMOylated peptides

Lyophilized peptides were dissolved in 10 ml ice-cold SUMO-IP buffer (50 mM MOPS, 10 mM Na_2_HPO_4_, 50 mM NaCl, buffered at pH 7.2) per 50 mg protein originally in the samples. Samples were clarified by centrifugation at 4250 *g* for 30 min at 4°C in a swing-out centrifuge with delayed deceleration. Samples were transferred to new tubes, after which 25 μl SUMO-IP beads was added per 50 mg protein originally in the samples. Samples were incubated at 4°C for 3 h in a rotating mixer, after which the beads were washed twice with ice-cold SUMO-IP buffer, twice with ice-cold PBS and twice with ice-cold MQ water. Upon each first wash with a new buffer, beads were transferred to a clean 1.5 ml LoBind tube (Eppendorf). To minimize loss of beads, all centrifugation steps were performed at 500 *g* for 3 min at 4°C in a swing-out centrifuge with delayed deceleration. Elution of SUMO peptides from the beads was performed by addition of 2 bead volumes of ice-cold 0.15% TFA, and performed for 30 min while standing still on ice, with gentle mixing every 10 min. The elution of the beads was repeated once, and both elutions were cleared through 0.45 μm spin filters (Millipore) by centrifuging at 12 000 *g* for 1 min at 4°C. The two elutions from the same samples were pooled after clarification. Next, samples were pH-neutralized by addition of 1/10th volume of 1 M Na_2_HPO_4_ and allowed to warm up to room temperature. Second-stage digestion of SUMOylated peptides was performed with 1 μg of Endoproteinase Asp-N (Roche). Digestion was performed overnight, at 30°C and shaking at 300 rpm, after which samples were frozen at −80°C until further processing.

### StageTip purification and high-pH fractionation of SUMO-IP samples

Preparation of StageTips (34), and high-pH fractionation of SUMO-IP samples on StageTip, was performed essentially as described previously (4). Quad-layer StageTips were prepared using four punch-outs of C18 material (Sigma-Aldrich, Empore™ SPE Disks, C18, 47 mm). StageTips were equilibrated using 100 μl of methanol, 100 μl of 80% ACN in 200 mM ammonium and two times 75 μl of 50 mM ammonium. Samples were thawed out, and supplemented with 1/10th volume of 200 mM ammonium, just prior to loading them on StageTip. The StageTips were subsequently washed twice with 150 μl of 50 mM ammonium, and afterward eluted as six fractions (F1–6) using 40 μl of 4, 7, 10, 13, 17 and 25% ACN in 50 mM ammonium. All fractions were dried to completion in LoBind tubes, using a SpeedVac for 2 h at 60°C, after which the dried peptides were dissolved using 10.5 μl of 0.1% formic acid.

### MS analysis

All samples were analyzed on EASY-nLC 1200 system (Thermo) coupled to a Q Exactive™ HF-X Hybrid Quadrupole-Orbitrap™ mass spectrometer (Thermo). For each run, 5 μl of sample was injected. Separation of peptides was performed using 15 cm columns (75 μm internal diameter) packed in-house with ReproSil-Pur 120 C18-AQ 1.9 μm beads (Dr. Maisch). Elution of peptides from the column was achieved using a gradient ranging from buffer A (0.1% formic acid) to buffer B (80% acetonitrile in 0.1% formic acid), at a flow of 250 nl/min. Gradient length was 80 min per sample, including ramp-up and wash-out, and an analytical gradient of 50 min. The buffer B ramp for the analytical gradient was as follows: F1: 13–24%, F2: 14–27%, F3–5: 15–30%, F6: 17–32%. The columns were heated to 40°C using a column oven, and ionization was achieved using a Nanospray Flex Ion Source (Thermo) with the spray voltage set at 2 kV, an ion transfer tube temperature of 275°C, and an RF funnel level of 40%. Full scan range was set to 400–1600 *m*/*z*, MS1 resolution to 60 000, MS1 AGC target to 3 000 000, and MS1 maximum injection time to 60 ms. Precursors with charges 2–6 were selected for fragmentation using an isolation width of 1.3 *m*/*z*, and fragmented using higher-energy collision disassociation (HCD) with normalized collision energy of 25. Precursors were excluded from re-sequencing by setting a dynamic exclusion of 60 s. MS2 resolution was set to 60 000, MS2 AGC target to 200 000, minimum MS2 GC target to 20 000, MS2 maximum injection time to 120 ms and loop count to 7.

### Analysis of MS data

All MS RAW data were analyzed using the freely available MaxQuant software, version 1.5.3.30 (35). All data were processed in a single computational run, and default MaxQuant settings were used, with exceptions specified below. For generation of the theoretical spectral library, the mouse FASTA database was downloaded from Uniprot on the 14 February 2020. The mature sequence of SUMO2 was inserted in the database to allow for detection of free SUMO. *In silico* digestion of theoretical peptides was performed with Lys-C, Asp-N and Glu-N, allowing up to 8 missed cleavages. Variable modifications used were protein N-terminal acetylation, methionine oxidation, peptide N-terminal pyroglutamate, Ser/Thr/Tyr phosphorylation (STY) and Lys SUMOylation, with a maximum of 3 modifications per peptide. The SUMO mass remnant was defined as described previously (4); DVFQQQTGG, H_60_C_41_N_12_O_15_, monoisotopic mass 960.4301, neutral loss b7-DVFQQQT, diagnostic mass remnants [b2-DV, b3-DVF, b4-DVFQ, b5-DVFQQ, b6-DVFQQQ, b7-DVFQQQT, b9-DVFQQQTGG, QQ, FQ, FQQ]. Label-free quantification was enabled, with ‘Fast LFQ’ disabled. Maximum peptide mass was set to 6000 Da. Stringent MaxQuant 1% FDR filtering was applied (default), and additional automatic filtering was ensured by setting the minimum delta score for modified peptides to 20, with a site decoy fraction of 2%. Second peptide search was enabled (default). Matching between runs was enabled, with a match time window of 1 min and an alignment window of 20 min. For protein quantification, the same variable modifications were included as for the peptide search. To further minimize false-positive discovery, additional manual filtering was performed at the peptide level. All modified peptides were required to have a localization probability of >75%, be supported by diagnostic mass remnants, be absent in the decoy database, and have a delta score of >40 in case SUMO modification was detected on a peptide C-terminal lysine not preceding an aspartic acid or glutamic acid. All multiply-SUMOylated peptides were discarded, unless the corresponding SUMO sites were also identified by singly-SUMOylated peptides. SUMO target proteins were derived from the ‘proteinGroups.txt’ file, and all post-filtering SUMO sites were manually mapped. Only proteins containing at least one SUMO site were considered as SUMO target proteins, and other putative SUMO target proteins were discarded.

### Calculation of SUMO density and equilibrium

SUMO density was calculated by dividing the total sum of all SUMO site intensity (in arb. units., corresponding to ion current) by the amount of total protein starting material (in mg). This calculation was performed for each replicate separately, and visualized as an average ± standard deviation. The mature sequence of SUMO-2 was included as a FASTA file in the MaxQuant search, to allow detection of free mature SUMO-2/3. For quantification of the SUMO equilibrium, the ‘modificationSpecificPeptides.txt’ MaxQuant output file was used, and all peptides modified by SUMO-2/3, and peptides derived from SUMO-2/3 itself, were considered. Modification of any SUMO family member by SUMO-2/3 was considered chain formation, with all other conjugation considered as global target modification. Peptides derived from SUMO-2/3 were sub-classed as internal, mature free SUMO-2/3, immature SUMO-2 or immature SUMO-3. Peptides ending in QQTGG (predominantly DVFQQQTGG) were considered as mature free SUMO-2/3. Peptides containing but not ending with QQTGG were considered as immature SUMO-2 (DVFQQQTGGVY) or immature SUMO-3 (DVFQQQTGGSASRGSVPTPNRCP).

### Western blotting

3T3-L1 cells were grown as described in ‘*Adipocyte culture, differentiation and treatment*’. Cells were washed with PBS prior lysis in ice-cold RIPA buffer (150 mM NaCl, 50 mM Tris-HCl, pH 7.5, 5 mM EDTA, 1% NP-40, 0.5% Na-deoxycholate, 0.1% SDS) freshly supplemented with protease inhibitor (Roche) and 20 mM of NEM. Lysates were sonicated 5 min (30 s on, 30 s off) then centrifuged 15 min at 14 000 rpm and 4°C. To eliminate lipids supernatants were applied to a RNeasy column and centrifuged at 10 000 rpm and 4°C for 1 min (Qiagen, 74104). The flow through was collected, and protein concentrations were assessed using the Bradford assay. About 30 μg of proteins were used for each western blot, and proteins were detected using anti-SUMO-2/3 (ab3742, Abcam) and anti-TBP (ab51841, Abcam) antibodies.

### Real-time PCR

Total RNAs were extracted as described in ‘*QuantSeq and SLAM-Sequencing sample preparation*’. RNAs were reverse transcribed with QuantiTect Rev. Transcription Kit (Qiagen, 205311). Real-time qPCR was performed using PowerUp™ SYBR™ Green Master Mix (Thermo Fisher Scientific, A25742) using primers designed for pre-mRNAs by targeting adjacent exon and intron. Scd1 forward: 5′-AAGTGAGGCGAGCAACTGAC; *Scd1* reverse: 5′-AACTCTCAACTTTCTCCTCCCG. Rgs2 forward: 5′-AGACCCGTTTGAGCTACTTCT; *Rgs2* reverse: 5′-CCTTTTGCTTGGCTTGGTTAG.

### Data analysis

The hierarchical clustering and heatmaps were generated using Perseus software v1.6.10.50 (36). For SUMO-2/3 proteomics, the label-free quantified (LFQ) value for four bio-replicates was averaged. The averaged value was log2 transformed, imputed with default setting, and normalized by row Z score transformation. For SLAMseq data, the normalized count for three bio-replicates was averaged. The averaged normalized count was log2 transformed and normalized by row Z score transformation. The row dendrogram was generated based on Euclidean distance and processed with *k*-means. Z score was used to generate cluster profiles in parallel.

GO analysis was performed using clusterProfiler R package (37). The statistical significance was specified as adjusted *P* value $ <$ 0.05. Redundant GO terms were simplified according to similarity measured with ‘Wang’ method.

The scatter plot was generated using Perseus software. Log2 transformed normalized counts were plotted. Meanwhile, Pearson correlation coefficient and P value were calculated.

Venn diagram was generated using VennDiagram R package (https://CRAN.R-project.org/package = VennDiagram).

For each single gene, Pearson correlation coefficient was calculated by comparing time-course profiles in different datasets in Excel. The density plot for the distribution of Pearson correlation coefficient was generated using EaSeq software v1.111 (28).

The ggplot2 R package v3.3.2 (https://ggplot2.tidyverse.org) was used to generate box, histogram, bar, dot and line plots in the study.

Integrative Genomics Viewer (38) was used for SLAMseq and ChIPseq data visualization with normalized bigWig files.

### Quantification and statistical analysis

For immunofluorescence data, Results are presented as mean ± SD. Between groups, statistical significance was calculated using unpaired, two tailed Student’s *t* tests using GraphPad Prism (v7). Non-significant: *P*$ >$ 0.05, *: *P* ≤ 0.05, **: *P* ≤ 0.01, ***: *P* ≤ 0.001, ****: *P* ≤ 0.0001. *P* values $ <$ 0.05 were considered as significant. For overrepresentation analysis, hypergeometric test was used for testing significance in R. *P* values were corrected by Benjamini–Hochberg method. For module analysis, ANOVA Post-Hoc (Dunnett) test was applied for testing the significance. Non-significant: *P*$ >$ 0.05, *: *P* ≤ 0.05, **: *P* ≤ 0.01, ***: *P* ≤ 0.001.

## RESULTS

### Inhibition of sumoylation results in lipoatrophy

To begin investigating the role of the sumoylation pathway during adipogenesis, we made use of the mouse 3T3-L1 cell model (39). Incubation of these cells in differentiation medium induces a well-defined adipocyte differentiation process, during which cells transition from a pre-adipocyte (PA) stage via a clonal expansion (CE) stage into mature adipocytes (MA). These transitions involve extensive regulation of gene expression and chromatin (40–42). We induced differentiation of 3T3-L1 cells either in absence or in presence of 500 nM of the selective SAE1/2 inhibitor ML-792, which is currently the molecule of choice to study mechanisms regulated by sumoylation because it does not inhibit other ubiquitin family pathways (43). In addition, long exposure to this concentration of ML-792 did not result in notable growth defects of 3T3-L1 cells (Supplementary Figure S1A,B). Western blotting of control cells with a SUMO-2/3 antibody revealed a progressive increase in SUMO conjugates during AD (Supplementary Figure S1C), indicating mobilization of the sumoylation pathway, whereas treatment with ML-792 almost completely prevented the accumulation of SUMO conjugates (Supplementary Figure S1C).

Next, we assessed the effect of ML-792 on fat accumulation using Bodipy reagents. Seven days after adipogenic induction there was a normal accumulation of lipid droplets in control cells (Supplementary Figure S1D), which is a key feature of adipocyte differentiation (39). In contrast, treatment with ML-792 resulted in lipoatrophy (Supplementary Figure S1D), which was characterized by numerous small lipid droplets (Supplementary Figure S1D–F), mirroring the recently reported phenotype of loss of *Ubc9* in mouse white adipose tissue (18). These data show that an active sumoylation pathway is required for efficient adipogenesis.

### Stage-specific total mRNA regulation of adipocyte differentiation

Given that sumoylation mostly targets nuclear proteins involved in chromatin and gene regulation (5), we hypothesized that ML-792 treatment resulted in lipoatrophy due to defects in adipogenic gene expression programs. To address this, we first assessed total RNA levels at different days of AD using QuantSeq (44) (Supplementary Figure S2A). Principal component analysis (PCA) revealed considerable changes in total mRNA levels between time points (Supplementary Figure S2B), which was further confirmed by the identification of 9426 differentially expressed mRNAs (Supplementary Figure S2C and Supplementary Table S1). Hierarchical clustering revealed mRNA modules with highest expression in pre-adipocytes (PA 1–3) or in mature adipocytes (MA 7–8). We also identified mRNA modules showing either very high or very low expression during clonal expansion (CE 4–6) (Supplementary Figure S2D,E and Supplementary Table S1). Although pathway enrichment did not show a single/strong function for each specific cluster, mRNAs encoding the known adipogenic inhibitors *Klf3* and *Klf10* (45) were downregulated upon induction (Supplementary Figure S2E). CE modules were specifically enriched for genes involved in mitotic cell cycle regulation and adipogenesis, such as *Top2a* and *Klf5* (45) (Supplementary Figure S2E,F). Finally, MA modules contained genes encoding specific adipogenesis markers, like *Pparg* and *Adipoq* (Supplementary Figure S2E,F). With this experiment we characterized steady state/total mRNA dynamics during adipogenesis, which showed that adipogenesis involves a complex regulation of total mRNA levels, which is consistent with previously reported RNA-seq data (41,46,47).

### The nascent transcriptome landscape of differentiating adipocytes

**Figure 1.**
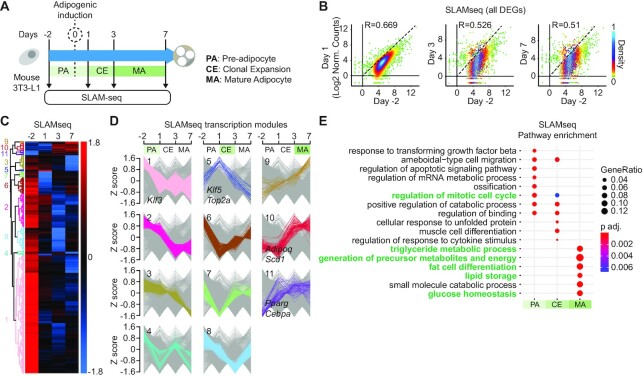
Characterization of nascent transcriptome dynamics during adipocyte differentiation. (**A**) Experimental layout of SLAMseq time-course experiments. (**B**) Scatter plots showing the nascent transcription levels for all SLAMseq time-course differentially expressed genes (DEGs) at each time point versus day −2. DEGs were identified based on statistical differences between conditions using a *P*-adj cutoff $ < 0.01$. The nascent transcription levels are presented as log2 transformed normalized counts. R, Pearson correlation coefficient. (**C** and **D**) Hierarchical clustering of SLAMseq DEGs. Clusters (C) are categorized in PA-, CE- and MA-specific modules (D); PA, pre-adipocyte; CE, clonal expansion; MA, mature adipocyte. *Z* score is calculated for log2 transformed normalized counts. Module numbers (D) are indicated on the left side of the heatmap in (C). (**E**) Pathway enrichment analysis of SLAMseq DEGs belonging to PA, CE and MA modules.

Although we observed clear differences in total mRNA levels at various stages of AD, delicate changes in gene transcription levels can be obscured by the large pool of background RNA and preclude the discovery of transcriptional regulators (48,49). Therefore, we used SLAMseq (44) to increase the temporal resolution of differential expression of nascent RNA during AD (Figure 1A). This revealed strong transcriptional differences between day −2, day 1 and day 3, whereas day 7 showed transcriptional levels similar to day 3 (Figure 1B and Supplementary Figure S3A). This indicates that most of the transcriptional rewiring during AD occurs until day 3, after which transcriptional output stabilizes as the adipocyte matures.

In total we identified 3705 SLAMseq DEGs during AD (Supplementary Table S2 and methods). Strikingly, in contrast to QuantSeq, SLAMseq analysis clearly showed a dramatic general downregulation of transcription upon adipogenic induction (Figure 1B and Supplementary Figure S2C). Comparison of common DEGs between SLAMseq and QuantSeq (Supplementary Figure S3B) revealed that this transcriptional shutdown translated into a reduction of mRNA levels starting from day 3 (Supplementary Figure S3C and examples in Supplementary Figure S3D). This indicates that although transcriptional repression of many genes occurs at induction of adipogenesis, many mRNAs remain available for several days. These data indicate significant divergence between total mRNA levels and actual transcription rates during adipogenesis.

Clustering analysis revealed 11 transcriptional modules (Figure 1C,D). As emphasized above, the vast majority of genes (3433 out of 3705, comprising modules 1–4 and 6–8) were transcribed in the PA stage and then downregulated upon adipogenic induction. Transcription of these genes reached minimum levels either during CE or at MA stage (Figure 1C,D; Supplementary Figure S3E; Supplementary Table S2), such as the anti-adipogenic gene *Klf3* (45). Interestingly, we noticed that a small number of 273 genes displayed increased transcription upon adipogenic induction. Transcription of a subset of these genes (module 5) was highly dynamic, showing a sharp increase during the transition from PA to CE, followed by strong downregulation as cells enter the MA stage. Closer inspection revealed that this module contained genes involved in mitotic cell cycle regulation and adipogenesis, such as *Top2A* and *Klf5* (45) (Figure 1C,D; Supplementary Figure S3E; Supplementary Table S2). Other genes (248) showed a more gradual increase in transcription levels and their expression remained high during later stages of AD (modules 9, 10 and 11), such as *Pparg* and *Adipoq*, both known to be highly expressed in MAs (Figure 1C,D; Supplementary Figure S3E; Supplementary Table S2).

Contrary to QuantSeq, pathway enrichment analysis of SLAMseq data revealed a partial overlap between terms in PA and CE (Figure 1E). These common terms notably included mitotic regulation of the cell cycle, indicating that transcriptional upregulation of cell cycle genes required for CE is one of the earliest events following adipogenic induction. In contrast, MA-specific transcription modules 9–11 showed a strong enrichment in genes involved in fat cell differentiation and mature adipocyte functions, including triglyceride metabolism, glucose homeostasis and lipid storage (Figure 1E).

We conclude that in contrast to what we observed at the total mRNA level using QuantSeq, adipogenic induction leads to a general transcriptional downregulation of most of the genes that are transcribed in pre-adipocytes. In parallel, the transcription of a relatively small number of strongly pro-adipogenic genes is upregulated and reaches maximum output in MAs (Figure 1C-E).

### SUMO promotes transcription of pro-adipogenic genes

**Figure 2.**
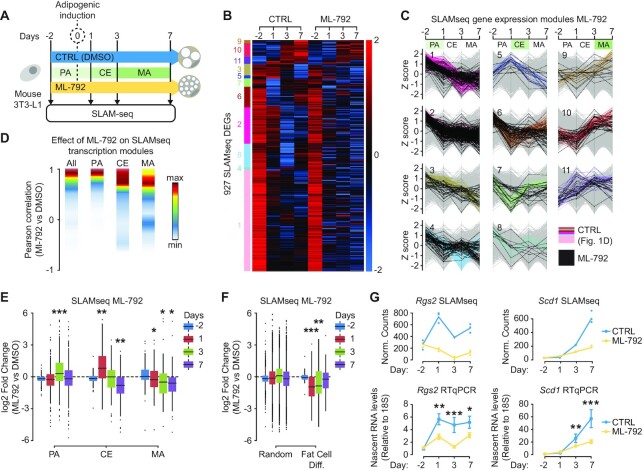
Sumoylation supports adipogenic genes transcription. (**A**) Experimental setup of time-course SLAMseq experiments in presence or absence of ML-792. CTRL: control cells were treated with DMSO. (**B**) Heatmap showing the effect of ML792 on time course SLAMseq DEGs (CTRL) identified in Figure 1D. DEGs were identified based on statistical differences between conditions (CTRL and ML-792) using a *P*-adj cutoff $ < 0.1$. (**C**) Effect of ML-792 on SLAMseq transcription modules revealed in Figure 1E. Z scores are calculated for log2 transformed normalized counts. Colored lines: SLAMseq time course DEGs (CTRL); Black lines: SLAMseq time course DEGs in presence of ML-792. Module numbers (C) are indicated on the left side of the heatmap in (B). (**D**) Density plot showing the effect of ML-792 on stage-specific SLAMseq transcription modules. Pearson correlation coefficients were calculated between the transcription profiles of time course DEGs in CTRL (DMSO) and ML-792 conditions. The *y* axis, presented as Pearson correlation coefficient, is segmented into 10 bins. The number of transcripts within each bin is presented as a color code. All: All ML-792 SLAMseq DEGs; PA, pre-adipocyte; CE, clonal expansion; MA, mature adipocyte. (**E** and **F**) Boxplots showing the effects of ML-792 on the transcription of SLAMseq DEGs in stage-specific modules (E) and genes belonging to the *fat cell differentiation pathway* GO term and randomly selected genes (F). The significance of mean comparison was determine using ANOVA followed by post hoc test. *: *P* ≤ 0.05, **: *P* ≤ 0.01, ***: *P* ≤ 0.001. PA, pre-adipocyte; CE, clonal expansion; MA, mature adipocyte. (**G**) Validation of selected sumoylation-dependent MA genes by qPCR. Upper panels: Loess regression lines showing SLAMseq transcription profiles of *Rgs2* and *Sdc1* in DMSO (CTRL) and ML-792-treated cells. The *y* axis is presented as normalized counts in SLAMseq. DMSO, blue; ML-792, yellow. Lower panels: Validation of *Rgs2* and *Sdc1* transcription profiles by RT-qPCR. Data are presented as mean ± standard deviation (ML-792 versus DMSO). DMSO, blue; ML-792, yellow. The significance of mean comparison was determine using ANOVA followed by post hoc test. *: *P* ≤ 0.05, **: *P* ≤ 0.01, ***: *P* ≤ 0.001.

Next, we studied the effect of the sumoylation pathway on the transcriptional landscape of differentiating 3T3-L1 cells by treating cells with ML-792 (Figure 2A). Differential expression analysis of ML-792 versus DMSO control samples revealed 5833 QuantSeq DEGs (9561 transcripts; Supplementary Table S3) and 905 SLAMseq DEGs (1334 transcripts; Supplementary Table S4). Whereas QuantSeq analysis did not detect significant directionality effects of ML-792 over time on total mRNA levels (Supplementary Figure S4A), SLAMseq revealed significant and global downregulation of transcription at days 1 and 7 and upregulation at day 3 (Supplementary Figure S4B), even when looking at individual time point DEGs and applying very stringent parameters that do not best-fit our experimental conditions or SLAMseq experiments (Supplementary Figure S4C). These data indicate that sumoylation inhibition alters transcription dynamics more significantly than it alters total mRNA dynamics. We therefore decided to focus our investigation on SUMO-regulated transcription using SLAMseq DEGs.

Among them, 636 DEGs (927 DE transcripts) were significantly regulated over the normal time course, while 407 genes were uniquely affected by ML-792 treatment (Supplementary Figure S4D). Regarding the 636 DEGs, pathway enrichment analysis revealed various processes including regulation of mitosis and fat cell differentiation, the latter being enriched beyond normal expectations (Supplementary Figure S4E,F). Hierarchical clustering revealed that sumoylation regulates the transcription of many genes located in PA, CE and MA modules (Figure 2B,C; Supplementary Table S4). Pearson correlation showed that PA modules were only mildly affected by ML-792 (Figure 2D and Supplementary Figure S4F), but that CE DEGs and in particular MA DEGs showed a strong deviation from transcription patterns observed in the control experiment (Figure 2D and Supplementary Figure S4F). This indicates that sumoylation is important for establishing the mature adipocyte program.

We next dissected the effect of sumoylation on AD modules by assessing transcriptional deregulation in each module for each time point. Interestingly, at day 3 many PA genes had failed to be downregulated when cells were treated with ML-792, indicating that the sumoylation pathway is important for repressing PA genes upon adipogenic induction (Figure 2E). We also found that ML-792 treatment caused many CE genes to remain strongly upregulated at day 1 but to become downregulated at day 7 (Figure 2E). This suggests that SUMO has a dual role in controlling these genes; it first represses these genes during entry into CE and then it reactivates them when cells enter MA stage. Finally, there was a substantial group of MA genes that failed to become activated in presence of ML-792 (Figure 2E), showing that sumoylation supports their transcription in mature adipocytes. More specifically, ML-792 treatment resulted in downregulation of MA-specific genes involved in fat cell differentiation starting from day 1 after adipogenic induction, while transcription of an equal number of randomly selected control genes was not affected (Figure 2F and Supplementary Table S4). The inhibitory effect of ML-792 on the transcription of adipogenic genes was validated for *Scd1* and *Rgs2* by qPCR (Figure 2G) (50,51).

Taken together, these data indicate that sumoylation has a complex and dynamic function in regulating the adipocyte differentiation program. In the early stages of AD, it is important for repression of PA genes and activation of CE genes, whereas in later stages it is required for maintaining high transcription levels of genes involved in fat cell differentiation and adipogenic function in mature adipocytes.

### SUMO supports the transcriptional identity switch from pre-adipocyte to mature adipocyte

**Figure 3.**
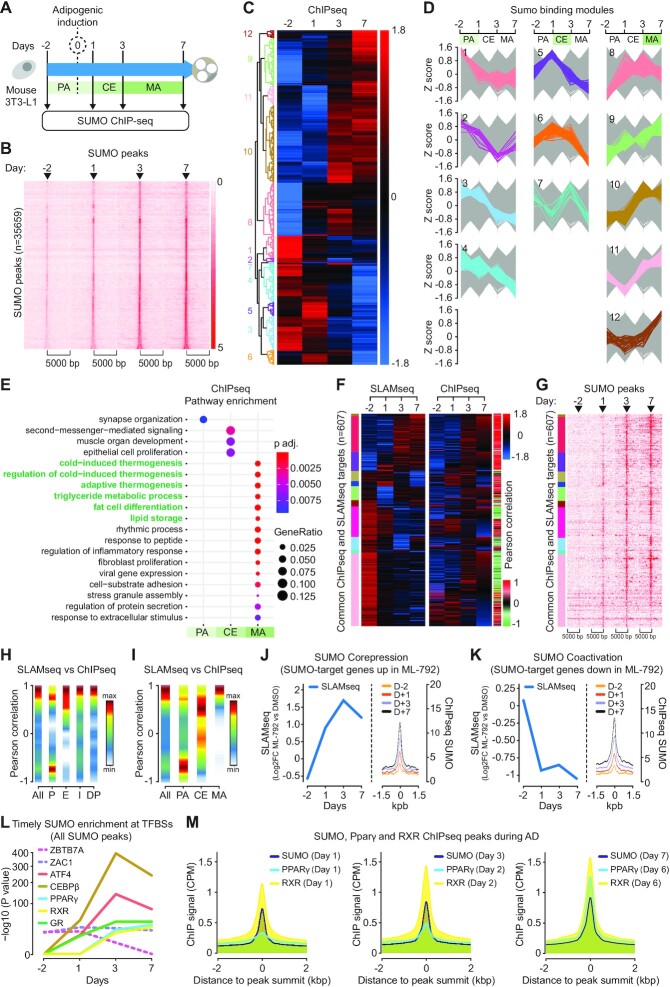
A dual role for SUMO at the chromatin. (**A**) Experimental setup of time-course ChIPseq experiment. (**B**) Heatmaps of SUMO occupancy within a 10 kb window centered on all SUMO peak summits. SUMO peaks are sorted by increasing size. The intensity represents log10 transformed FPKM. (**C** and **D**) Hierarchical clustering of differentially enriched SUMO peaks during the time course (C). Clusters are further categorized to 3 stage-specific modules: PA, CE and MA (D). Z scores are calculated for log2 transformed normalized tags. PA, pre-adipocyte; CE, clonal expansion; MA, mature adipocyte. Module numbers (D) are indicated on the left side of the heatmap in (C). (**E**) Pathway enrichment analysis of SUMO target genes belonging to PA, CE and MA modules. (**F**) Heatmaps showing the SLAMseq time course clusters (Figure 1D) and corresponding SUMO ChIPseq peaks. Pearson correlation coefficients between transcription levels and SUMO occupancy of genes are indicated by gradient color. (**G**) Heatmaps of SUMO occupancy at the genes within a 10 kb window centered on peak summits. SUMO peaks are sorted in the same order as in (F). The intensity represents log10 transformed FPKM. (**H** and **I**) Distributions of Pearson correlation coefficients showed in (F) in groups categorized base on genomic locations of SUMO peaks (H) and AD modules (I). All: all SUMO peaks, P: promoter-TSS, E: exon, I: intron, DP: distant promoter. PA, pre-adipocyte; CE, clonal expansion; MA, mature adipocyte. The *y* axis, presented as Pearson correlation coefficient, is segmented into 10 bins. The number of transcripts within each bin is presented as a color code. (**J**) The corepressor function of SUMO. Left panel: Profile (Loess regression lines) of SLAMseq DEGs that are upregulated in presence of ML-792. The result is presented as log2 fold change between DMSO (CTRL) and ML-792-treated cells. Right panel: Mean profile of SUMO occupancy (ChIPseq coverage) within a 3 kb window at genes upregulated in ML-792-treated cells. (**K**) The coactivator function of SUMO. Left panel: Profile (Loess regression lines) of SLAMseq DEGs that are downregulated in presence of ML-792. The result is presented as log2 fold change between DMSO (CTRL) and ML-792-treated cells. Right panel: Mean profile of SUMO occupancy (ChIPseq coverage) within a 3 kb window at genes downregulated in ML-792-treated cells. (**L**) Waves of enrichment of adipogenic transcription factor binding sites (TFBS) at SUMO peaks during AD. Dashed lines: Examples of enrichment of non-adipogenic TFBSs. All SUMO peaks were analyzed in an unbiased manner. *P* value accounts for motif enrichment. (**M**) Mean profiles of SUMO, PPARγ and RXR ChIPseq signals within a 4 kb window centered on peak summits during AD. CPM (counts per million reads mapped) was used for comparing multiple ChIPseq data. PPARγ and RXR ChIPseq data originate from a previously published study (41).

Previous studies showed that the SUMO-2/3 chromatin landscape is highly dynamic in response to various signals (6–9,12). To gain insight into the dynamics and location of SUMO-2/3 at the chromatin during AD we performed ChIPseq experiments using an anti SUMO-2/3 antibody (Figure 3A). For the sake of simplicity and better readability, SUMO-2/3 will be simply referred to as SUMO in the rest of this study. PCA revealed good correlation between biological replicates and strong variation between time points (Supplementary Figure S5A). We identified 35 659 SUMO peaks (Supplementary Table S5), which were primarily located at transcription units, especially at promoters (Supplementary Figure S5B). Strikingly, both the number and intensity of SUMO peaks increased after induction of adipogenesis (Figure 3B). About 1850 significant differential SUMO binding sites were identified over the time course (Supplementary Table S5). Pathway enrichment analysis showed that SUMO peaks are overrepresented at genes involved in fat cell differentiation and genes with hallmark MA functions, such as lipid metabolism (Supplementary Figure S5C).

Hierarchical clustering identified SUMO binding modules with the highest ChIP signals at PA, CE and MA stages (Figure 3C,D and Supplementary Table S5). PA modules 1–4 contained 544 SUMO peaks of which the intensity decreased upon adipogenic induction. These peaks were assigned to a heterogeneous set of genes that did not show significant pathway enrichment (Figure 3E). The same was observed for CE modules 5–7 that contained 170 peaks with highest intensity at day 1 or 3 (Figure 3E). Finally, MA modules 8–12 contained 1136 peaks with highest intensity in MAs and a very strong enrichment for genes involved in adipogenic functions as well as adaptive thermogenesis (Figure 3E). These data indicate that chromatin-bound SUMO may support adipocyte function and metabolism via transcriptional regulation.

By comparing the SLAMseq transcription modules identified in Figure 1D with SUMO ChIPseq data, followed by hierarchical clustering, we found that distinct peaks of SUMO were present both at repressed genes and at activated genes upon induction of adipogenic differentiation (Figure 3F,G and Supplementary Table S5). Pearson scoring revealed a general positive correlation between transcription and the presence of SUMO (Figure 3H,I), which was especially true for exons, introns and distant promoters (Figure 3H). However, the presence of SUMO at promoter regions both positively and negatively correlated with transcription (Figure 3H). Most PA genes, including the AD inhibitor *Klf10*, appeared to be repressed in presence of SUMO upon induction of adipogenic differentiation (Figure 3I and Supplementary Figure S5D). In clear contrast, the presence of SUMO at CE genes and in particular at MA genes, such as the adipogenic gene *Fabp4*, showed a positive correlation with transcription (Figure 3I and Supplementary Figure S5D).

Based on these findings, we hypothesized that adipogenic stimulation on the one hand leads to increased sumoylation of transcription factors to inhibit transcription of PA genes, while on the other hand it increases transcription of MA genes. We integrated ML-792 SLAMseq data with CTRL SLAMseq and ChIPseq datasets by hierarchical clustering (Supplementary Figure S5E and Supplementary Table S5). This revealed two main categories of SUMO-target genes. In the first category, SUMO-target genes were upregulated upon treatment with ML-792, which indicates a corepressor function of SUMO (Figure 3J, Supplementary Figure S5F and Supplementary Table S5). In the second category, SUMO-target genes were downregulated upon treatment with ML-792, which indicates a coactivator function of SUMO (Figure 3K, Supplementary Figure S5F and Supplementary Table S5). Generally, genes activated by SUMO are involved in fat cell differentiation and carbohydrate metabolism, whereas genes repressed by SUMO are not related to adipogenesis (Supplementary Figure S5G).

We conclude that (i) chromatin-bound SUMO has a dual role during AD by repressing PA genes while promoting MA genes and (ii) that SUMO plays an instrumental role in the transcriptional identity switch from pre-adipocyte to mature and functional adipocyte.

### Adipogenic differentiation is associated with waves of SUMO on chromatin

To identify potential transcription factor binding sites (TFBS) enriched for SUMO, we performed an unbiased motif search at all SUMO ChIPseq peaks. We did not find a significant enrichment for adipogenic TFBS sequences at day −2 (Figure 3L and Supplementary Table S6). However, we observed significant recruitment of SUMO at critical adipogenic TFBSs over time after stimulation of adipogenic differentiation (Figure 3L; Supplementary Figure S5H and Supplementary Table S6). A first wave of SUMO was detected at binding sites for CEBPβ, GR and ATF4 shortly after adipogenic induction from day 1 until day 7. A second wave of SUMO occurred at PPARγ and RXR response elements, starting on day 3 and lasting until day 7 (Figure 3L and Supplementary Figure S5H). In parallel, the presence of SUMO at non-adipogenic TFBSs, like at the binding sites for the repressor ZBTB7A and the inhibitor of cell proliferation ZAC1, was either reduced or remained unchanged upon adipogenic induction.

We then focused on SUMO peaks found at SLAMseq DEGs. At these genes, the binding of SUMO at adipogenic TFBSs was more significant than at any other non-adipogenic TFBS (Supplementary Figure S5I and Supplementary Table S6). These data indicate that the increasing presence of SUMO at the chromatin during AD is specifically directed toward adipogenic TFBSs.

To validate these findings, we compared our SUMO ChIPseq data with published PPARγ/RXR ChIPseq (41) and found that there was a very significant and progressive timely overlap between the location of SUMO, PPARγ and RXR peaks during AD (Figure 3M and Supplementary Table S7). Notably, SUMO recruitment to PPARγ/RXR TFBSs followed the known timeline of recruitment of these TFs during AD (41,52).

These data indicate that the increase in SUMO levels at genes during AD occurs at very specific adipogenic TFBS like CEBPβ, GR and PPARγ/RXR.

### Site-specific characterization of the SUMOylome during adipocyte differentiation

**Figure 4.**
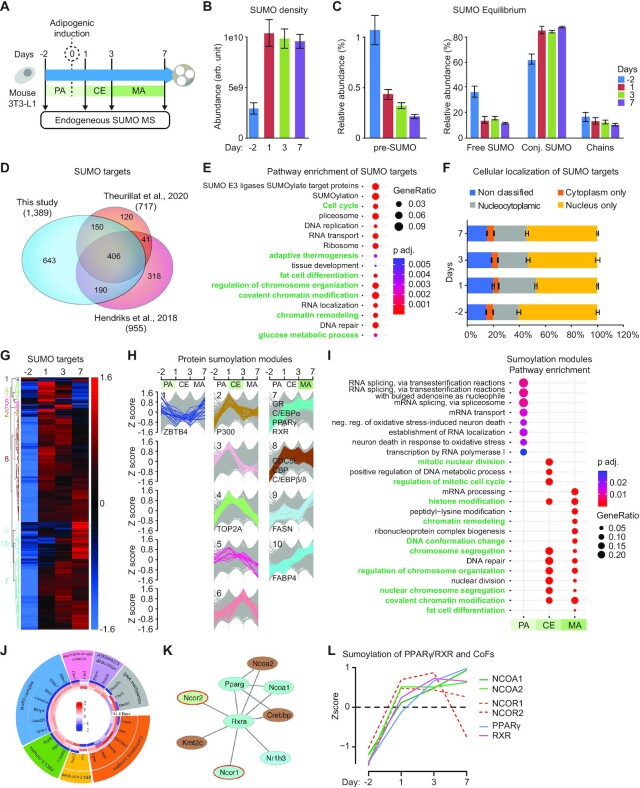
Site specific characterization of the endogenous SUMOylome of differentiating adipocytes. (**A**) Experimental setup of time-course endogenous SUMO-2/3 proteomics. (**B**) Quantification of total SUMO-2/3 density during adipogenesis (normalized to input amount; *n* = 4). (**C**) Quantification of the SUMO2/3 equilibrium during adipogenesis. Left: Fraction of total SUMO existing as immature SUMO (pre-SUMO). Right: Fraction of total SUMO existing as free SUMO, SUMO conjugated to target proteins and SUMO chains. The *y* axis is presented as relative abundance to total SUMO-2/3 density at each time point (*n* = 4). (**D**) Venn diagram of SUMO targets identified in this study and two recently published studies that used endogenous detection of sumoylated sites. (**E**) Pathway enrichment analysis of SUMO targets identified by mass spectrometry. (**F**) The percentage of all SUMO targets located in the nucleus, the cytoplasm or both during adipogenesis. (**G**) Hierarchical clustering of SUMO targets (G). Clusters are categorized in three sumoylation modules (H): PA, pre-adipocyte; CE, clonal expansion; MA, mature adipocyte. Module numbers (H) are indicated on the left side of the heatmap in (G). (**I**) Pathway enrichment analysis of proteins belonging to PA, CE and MA sumoylation modules. (**J**) Circular plot showing the representative SUMO targets involved in epigenetic and chromatin remodeling complexes at each time point during adipocyte differentiation. The dynamics of sumoylation intensity during AD is presented in the circular heatmap. *Z* scores are calculated for log2 transformed label-free quantified (LFQ) values. (**K**) STRING network of SUMO targets within the PPARγ/RXR complex. Repressors are outlined with a red line. (**L**) Loess regression plot of sumoylation intensity for selected SUMO targets in PPARγ/RXR complex. Solid line: PPARγ, RXR and co-activators; dashed line: co-repressors. Z scores are calculated for log2 transformed label-free quantified (LFQ) values.

Our ChIPseq data show that SUMO binds to specific TFBSs during AD. To identify adipogenesis-specific SUMO-2/3 substrates, we carried out site-specific characterization of the endogenous SUMOylome by mass spectrometry during AD (Figure 4A) (4). PCA of SUMO-modified lysine residues of four independent experiments demonstrated high reproducibility between replicates and considerable differences between the time points (Supplementary Figure S6A,B). Across all time points, fractions and biological replicates, we identified 5230 SUMO-modified peptides that mapped to 3706 unique SUMO sites. Out of all SUMO sites, 3137 (∼85%) could be quantified in quadruplicate (Supplementary Table S8).

In accordance with SUMO western blots (Supplementary Figure S1C), MS experiments showed that the overall density of SUMO increases upon adipogenic induction (Figure 4B). Furthermore, when we analyzed the SUMO equilibrium, i.e. the distribution of SUMO across the entire system and whether it exists in a free or in a conjugated form, we found a larger portion of immature as well as free SUMO prior to induction of differentiation (Figure 4C). Upon adipogenic induction the consumption of free SUMO increased to reach near-maximum levels at the later time points (Figure 4C). We then correlated our whole transcriptome data (QuantSeq) to the sumoylome of differentiating adipocytes. Knowing that the transcriptome has a predictive value for determining the expected proteome (15,53), we did not find any correlation between the expression of proteins and their sumoylation throughout adipogenesis (Supplementary Figure S6C,D). Although we cannot exclude the possibility that changes in individual proteins levels are in part responsible, these data indicate that the increased detection of SUMO sites during AD is not due to a global increase in the abundance of proteins. Therefore, changes in the levels of the mRNA or the protein do not predict whether this protein is a SUMO target, which is consistent with a previous report (15). We did not detect significant alteration of the formation of SUMO chains during AD (Figure 4C).

In total, we identified 1389 SUMO target proteins, of which 1250 could be quantified in quadruplicate with high reproducibility (Supplementary Figure S6E and Supplementary Table S8). This is presently the largest number of SUMO targets identified using this methodology (Figure 4D) (4,15). In addition, despite a significant overlap of SUMO targets between our study and previous ones, we identified a very large number (643) of adipocyte-specific SUMO targets (Figure 4D), which indicates that adipocyte differentiation heavily relies on sumoylation. Pathway enrichment analysis showed that these targets are involved in chromatin regulation, transcription, chromosome maintenance and cell cycle progression (Figure 4E and Supplementary Table S8), with specific enrichment of pathways that are critical for adipose tissue development, function, adaptive thermogenesis and brown cell differentiation (Figure 4E and Supplementary Table S8). Remarkably the majority of SUMO targets (∼80%) are nuclear or nucleocytoplasmic proteins while only ∼5% are cytoplasmic (Figure 4F and Supplementary Table S8), which is consistent with previous studies (5).

Hierarchical clustering of the SUMO targets underlined the sharp remodeling of the SUMOylome occurring after adipogenic induction (Figure 4G and Supplementary Table S8). Only very few proteins (21) were highly sumoylated at PA stage; a notable example being the transcriptional repressor ZBTB4 (Figure 4G,H and Supplementary Figure S6F). 174 out of 1250 SUMO targets became highly sumoylated during CE (Figure 4G,H). These proteins are involved in mitotic chromosome and centromere regulation, nucleosome modification and DNA methylation, and include TOP2A, CDCA5, P300, CENPS, DNMT1, HDAC2 and HDAC4 (Figure 4H–J and Supplementary Figure S6F), supporting the idea that SUMO is important for mitotic events and chromatin regulation during CE. The 1055 MA-specific targets were enriched for proteins critical for transcriptional silencing like polycomb and DNMT1, chromatin remodeling like NuRD, histone modifications like CBP and TRIM28 and fat cell differentiation like the pro-adipogenesis TFs GR, CEBPα/δ, PPARγ and RXR (Figure 4H–J and Supplementary Figure S6F). These targets also include a few proteins involved in fat metabolism, such as FABP4 or FASN (Supplementary Figure S6F). Thus, sumoylation of adipogenic TFs clearly correlates with MA stage and indicates that SUMO supports adipogenic function at the transcription level.

Next, we mapped the interaction network of sumoylated TFs using STRING network analysis (54). Integration of the STRING network with sumoylation modules revealed that two TFs/CoFs were sumoylated in PA, 12 in CE and 96 in MA modules (Supplementary Figure S6G). Interestingly, only nine proteins were classified as transcriptional repressors (Supplementary Figure S6G). A major interaction node was centered on CDC5l and interactors, which are sumoylated early during AD and in MAs (Supplementary Figure S6G and Supplementary Table S8). Interestingly, CDC5l is involved in mitotic progression and has been associated with fast cell cycle progression during the early phase of stem cell reprogramming (55), suggesting that sumoylation of CDC5l and its interactors contributes to cell cycle progression during CE. Another major node is centered on the histone acetyltransferase CREBBP (CBP), which is an interaction partner and transcriptional co-activator for many TFs, including the nuclear receptors PPARγ/RXR (Figure 4H and Supplementary Figure S6F,G). PPARγ/RXR response elements are strongly occupied by SUMO in MA stage (Figure 3L,M), consistent with a model in which sumoylation of PPARγ/RXR and coactivators is important for transcriptional regulation in mature adipocytes. Indeed, PPARγ/RXR co-repressors may not contribute as much as co-activators to the SUMO signal detected at PPREs because NCOR1 and NCOR2 sumoylation is lower in MA than in CE stage whereas sumoylation of NCOA1, NCOA2, NURD and PPARγ/RXR reached maximum levels in mature adipocytes (Figure 4K,L).

These data indicate that the timely sumoylation of mitotic, chromatin and transcriptional regulators supports the CE and MA stages of AD. Furthermore, our data strongly suggest that sumoylation of chromatin bound PPARγ/RXR and their CoFs supports adipogenic transcription.

### Sumoylation supports transcription of PPARγ/RXR target genes in mature adipocytes

**Figure 5.**
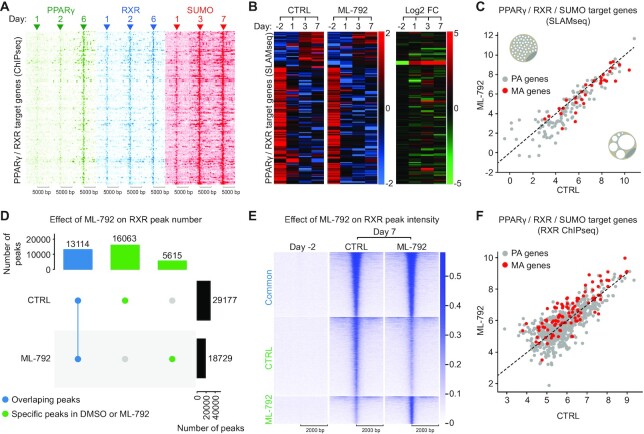
PPARγ, RXR and SUMO promote adipogenic genes transcription. (**A**) Heatmaps of PPARγ, RXR and SUMO occupancy within a 10 kb window centered on common peak summits of PPARγ/RXR/SUMO common target genes. The intensity of ChIPseq signals is presented as log10 transformed Fragments Per Kilobase of transcript per Million mapped reads (FPKM). SUMO peaks are sorted according to the order of the rows in (B). (**B**) Hierarchical clustering of PPARγ/RXR/SUMO common target genes identified in (A). *Z* score is calculated for log2 transformed normalized counts. (**C**) Scatter plot showing the global downregulation of transcription of PPARγ/RXR/SUMO common target genes upon ML-792 treatment at day 7. The nascent transcription levels (SLAMseq) are presented as log2 transformed normalized counts. In normal conditions SUMO supports the transcription of PPARγ-RXR target genes to promote the formation and function of mature adipocytes (MA) (lower right adipocyte). Downregulation of MA-specific SUMO target genes by ML-792 leads to lipoatrophy (upper left adipocyte). (**D**) Upset plot presenting the number of overlapping peaks when comparing RXR ChIPseq in DMSO (CTRL) and in ML-792 at day 7 post adipogenic induction. (**E**) Heatmap showing the effect of ML-792 on the intensity of overlapping, CTRL and ML-792 peaks at day 7 post adipogenic induction. (**F**) Scatter plot showing the alteration of RXR occurrence at PPARγ/RXR/SUMO common target genes in (A–C) upon ML-792 treatment. Gray dots: PA genes; Red dots: MA genes. RXR occurrence (ChIPseq) is presented as log2 normalized Tags.

Our data indicate that the SUMO peaks detected at PPARγ/RXR binding sites (Figure 3 and Supplementary Figure S3) are due to the presence of sumoylated TFs and co-activators, including PPARγ/RXR itself (Figure 4). We integrated the SUMO MS, SUMO SLAMseq, SUMO ChIPseq, and PPARγ/RXR ChIPseq datasets and focused on PPARγ/RXR target genes of which the regulation is critical during AD (41). We identified 143 DEGs, both normal time-course DEGs and ML-792 DEGs, at which SUMO perfectly overlapped with PPARγ/RXR (Figure 5A and Supplementary Table S9). Note that these genes are adipogenic genes (Supplementary Figure S7A) that are either repressed (PA genes) or activated (MA genes) by adipogenic stimulation (Figure 5B and Supplementary Figure S7B). PPARγ/RXR became sumoylated upon adipogenic induction, which was subsequently followed by an increased presence of SUMO at PPARγ/RXR response elements (Supplementary Figure S7B; *example of Scd1 gene in*Supplementary Figure S7C). Importantly, treatment with ML-792 led to the downregulation of PA genes and upregulation of MA genes at day −2, whereas MA genes were strongly downregulated at day 7 (Figure 5B,C and Supplementary Figure S7B,C).

We then hypothesized that the transcriptional deregulation in ML-792-treated cells was linked to an alteration of the chromatin. To address this, we analyzed the chromatin occurrence of RXR in absence or presence of ML-792 using ChIPseq at day −2 and day 7. Consistent with previous studies, RXR was weakly detected at day −2 and strongly detected at day 7 post adipogenic induction (Supplementary Figure S7D) (41). At day 7, differential peak analysis revealed a strong divergence between CTRL cells and ML-792 treated cells (Supplementary Figure S7E). First, we detected 10 448 less RXR peaks in ML-792-treated cells (18 729) than in DMSO-treated cells (29 177) (Figure 5D and Supplementary Table S10). Second, 5615 RXR peaks were specifically detected in ML-792-treated cells but not in control cells (Figure 5D and Supplementary Table S10). Third, although there are significant variations within each cluster, common DMSO/ML-792 peaks were overall not affected by sumoylation inhibition while the intensity of DMSO-specific peaks and ML-792-specific peaks decreased and increased, respectively (Figure 5E and Supplementary Figure S7F). These data show that while many RXR peaks remain unaffected, inhibition of sumoylation led to a significant decrease of RXR occurrence at the chromatin as well as the appearance of ML-792-specific peaks of RXR.

Pathway enrichment analysis of the genes belonging to each of the three clusters described in Figure 5E revealed the enrichment of numerous pathways and an important overlap between clusters (Supplementary Figure S7G). This is due to the large number of RXR targets in each cluster. However, our results show that the ML-792 cluster is enriched for genes involved in fat cell differentiation, suggesting that inhibition of sumoylation enhanced the occurrence of RXR at adipogenic genes.

We then addressed the link between alteration of RXR occurrence at the chromatin and transcriptional deregulation in ML-792 by comparing RXR ChIPseq and SLAMseq. Despite significant alterations of RXR levels at the chromatin between clusters in Figure 5E, we did not detect a strong global effect on transcription of RXR target genes (Supplementary Figure S7H). This is not surprising since RXR is not the only sumoylated TF regulating these genes. Nonetheless, we observed that the increase of RXR occurrence in the ML-792 cluster correlated with a mild yet significant increase of the transcription of corresponding RXR target genes (Supplementary Figure S7H).

On the other hand, at SUMO/RXR/PPARγ target genes, which are downregulated by ML-792 (see Figure 5A–C and supplementary Figure S7B), transcriptional inhibition was linked to the deregulation of RXR recruitment (Figure 5F). Specifically, at MA genes like *Scd1*, the occurrence of RXR was increased in presence of ML-792 (Figure 5F and Supplementary Figure S7I).

These data indicate that the regulation of PPARγ/RXR target genes in response to adipogenic stimulation requires the activity of the sumoylation pathway. Inhibiting the sumoylation process down-regulates these genes, which is linked to alteration of the occurrence of RXR at the chromatin and resulted in lipoatrophy (Supplementary Figures S1B and S5C).

## DISCUSSION

Evidence that the sumoylation pathway supports adipose tissue formation and function has been provided by previous studies (16–21). The majority of these studies have concluded that sumoylation generally inhibits adipogenic transcription factors. This view of sumoylation has been dominant for many years, but recent systematic large-scale studies have revealed that SUMO not only represses but can also promote transcription, depending on the cellular system and the physiological context (2,56). Systematic analysis of the dynamics of SUMO-regulated gene expression in differentiating adipocytes has thus far not been performed. In the current study we have shown that adipogenesis involves a dramatic mobilization of the sumoylation pathway, a dynamic reorganization of the sumoylome at transcription units, and that SUMO is important for establishment and robustness of the adipogenic transcription program to enforce the identity shift from pre-adipocyte to mature adipocyte.

As previously published in other cellular systems, the abundance of mature mRNAs compared to nascent RNAs and compensatory mechanisms that increase mRNA stability upon transcriptional inhibition may occlude transcriptional changes that could lead to the discovery of factors that regulate transcription (48,49). To investigate adipogeneic transcription, we characterized nascent transcriptome dynamics during adipogenesis using SLAMseq. Interestingly, comparison of SLAMseq data with total mRNA dynamics (QuantSeq) revealed important deviations. As previously observed, total mRNA dynamics featured gene expression modules involving large numbers of genes and intricate dynamics (41,46). In contrast, SLAMseq revealed a severe transcriptional switch from PA to MA stages: PA genes (92%) are repressed and MA (also some CE) genes (8%) are induced. Regulation of transcription upon adipogenic induction is thus much sharper than what can be observed using more classical RNA-seq methods or QuantSeq. Our data also reveal a delay between transcriptional regulation and alteration of total mRNA levels. Finally, the effect of sumoylation inhibition on nascent transcripts (SLAMseq) was more pronounced than on total mRNA levels (QuantSeq). This is consistent with the mild alteration of total mRNA levels by ML-792 that was previously observed in another cellular system using classical mRNAseq (43). The divergence between transcription and total mRNA data might be due to compensatory mechanisms regulating mRNA stability, which could also play an important role during adipogenesis. The investigation of the biological significance of the deviation between transcription and mRNA stability during adipogenesis will be the subject of our follow-up studies.

The presence of SUMO on chromatin is generally believed to be associated with the establishment of repressive chromatin (9,10,12,13,15). Indeed, we found that SUMO has a predominantly repressive function at the early stages of AD, when 92% of PA genes are repressed; treatment with ML-792 results in unscheduled activation of these genes. The mechanism by which SUMO represses these genes is the focus of our ongoing studies. Sumoylation of transcriptional activators could inhibit their transactivation activity via the recruitment of corepressors that close the chromatin, as has recently been shown for the glucocorticoid receptor in HEK-293 cells (6). Alternatively, our MS data indicate that modification by SUMO may result in the recruitment or activation of chromatin silencers that establish and maintain PA gene silencing in mature adipocytes, such as the heterochromatin polycomb complex or DNMTs (9,15).

Of note, the sumoylation pathway also has a distinct positive effect on transcription of a subset of genes. Here, adipogenic induction results in increased levels of SUMO to genes known to be important for the MA state, and the presence of SUMO at these genes correlated positively with their transcription. We observed an unexpected dynamic involvement of SUMO in regulation of transcription during AD, which appears to involve two waves of sumoylation at the promoter of MA genes. The early wave mainly involves ATF4, CEBPβ and GR binding sites. Interestingly, CEBPβ and GR have been shown to be critical for adipogenic transcription in the first hours after adipogenic induction (40,52). These proteins (and also CEBPδ) also rank amongst the earliest and top SUMO targets in our sumoylome study. Given that sumoylation of GR regulates its transactivation activity in other cellular types (6), our data suggest that sumoylation of these factors regulates the transcription of immediate adipogenic genes. GR and cEBPs bind the chromatin almost immediately after adipogenic induction (57), but we started collecting samples 24 h after adipogenic induction. This is a limitation of our study that prevented a precise investigation of the regulation of these early factors by SUMO (as well as other relevant SUMO targets), which will be the scope of another study using dedicated experimental conditions.

The second wave of sumoylation hits PPARγ/RXR response elements (PPRE) and peaks at the MA stage. It is possible that multiple components of the PPARγ/RXR complex become sumoylated because our MS data show that the PPARγ/RXR coactivators Ncoa1, Ncoa2 and CBP and chromatin remodeling complexes like NuRD are all SUMO targets. Exactly how SUMO regulates these proteins to activate transcription remains to be established but could involve stabilization of protein–protein interactions within the complex or regulation of chromatin occurrence.

Given that the presence of RXR at the chromatin matches gene expression and genome architecture dynamics during adipogenesis (41,42), sumoylation of RXR and other chromatin-bound SUMO targets could support these critical aspects of adipogenesis. Our ChIPseq experiments indicate that sumoylation supports the occurrence of RXR at the chromatin in three ways. First, ML-792 caused the disappearance of about 35% of RXR peaks and second, the appearance of 5616 specific peaks. These data are consistent with the previously reported role of sumoylation in altering the capacity and the specificity of transcription factors to bind the DNA (9,11,58). Third, ML-792 triggered an increase of RXR occurrence at PPAR/RXR/SUMO MA target genes, which correlated with transcriptional inhibition. This suggests that sumoylation promotes the turnover of RXR at these genes to support adipogenic transcription. A rapid turnover of nuclear receptor is indeed required to sustain high levels of transcription (59–61). Interestingly, a slower turnover of TFs upon sumoylation inhibition or using non-sumoylatable versions (KR mutants) of transcription factors Rap1 and GR in yeast and mammals, respectively has previously been documented (6,7). In the case of Rap1, this led to transcriptional repression while in the case of GR it led to activation. This suggests that sumoylation regulates the turnover of TFs that is required during both activation and repression of transcription (2). However, how sumoylation regulates the occurence of RXR and other TFs at the chromatin remains an open question. Sumoylation could regulate RXR protein networks and accessibility of RXR to nuclear receptor responsive elements (6–8). We did not address this mechanism in our study. Nonetheless, we have found 3706 sumoylation sites and 1389 unique SUMO targets during adipogenesis using mass spectrometry. Among these, we identified specific sumoylated lysines of RXR (K113,123,250) as well as sumoylated lysines of 109 other transcriptional regulators, including GR, PPARγ, P300, NCoR, NCoA and cEBPs. This should be an excellent starting point for in-depth mechanistic studies, for instance using ChIPseq and ChIP-SICAP, as it has recently been done for GR (6).

Our findings that sumoylation of PPARγ/RXR and its coactivators supports the adipogenic transcription program is in apparent contrast with previous reports showing that sumoylation inhibits PPARγ activity (62–64). However, these previous studies mostly addressed the regulation of PPARγ by SUMO-1 rather than SUMO-2/3 using single PPARγ mutants and a small selection of model genes (65). In contrast, our comprehensive genome-wide approach clearly shows that inhibition of the sumoylation pathway strongly reduces the expression of PPARγ/RXR target genes. Thus, the physiological relevance of the sumoylation pathway during AD is that it has a net overall positive effect on the PPARγ/RXR transcription program, which is also consistent with the lipoatrophy phenotype that we and others have observed upon inhibition of sumoylation (16,18). A similar apparent discrepancy has been observed in yeast, where one study reported that sumoylation of specific lysines in the RNA polymerase III complex activates the enzyme (8), whereas a second study found that sumoylation of another set of lysines of components of the complex resulted in the opposite effect (66). By expressing a mutant form of the E2 conjugase UBC9, which results in strongly reduced activity of the sumoylation pathway, it was subsequently shown that under optimal growth conditions the net overall effect of sumoylation is to promote activity of the complex (8). This example shows that different sumoylation sites can have opposing effects on the activity of a protein complex, and that the cell likely targets specific sumoylation sites depending on the cellular state. We speculate that the same may be the case for the PPARγ complex. Depending on environmental cues, cells use different sumoylation sites and SUMO isoforms to finetune the activity of the PPARγ complex, resulting either in its activation or inhibition; however, we believe that during normal AD the overall effect of sumoylation on PPARγ/RXR is to activate the complex. Clearly, more experiments are required to dissect the exact mechanism by which sumoylation regulates the PPARγ/RXR complex.

In conclusion, our study provides novel insight into the role of the sumoylation pathway during dynamic changes in transcriptional and chromatin regulation that occur during adipocyte differentiation, and also provides a wealth of data that should be of interest for researchers working in the fields of sumoylation, transcription and adipocyte differentiation.

## DATA AVAILABILITY

The SLAMseq, QuantSeq, SUMO ChIPseq and RXR ChIPseq data have been deposited to the Gene Expression Omnibus with accession number GSE167222.

The accession numbers for previously published PPARγ and RXR ChIPseq data are GSM340795, GSM340796, GSM340799, GSM340801, GSM340802 and GSM340805 (41).

The mass spectrometry proteomics data have been deposited to the ProteomeXchange Consortium via the PRIDE partner repository with the dataset identifier PXD024144.

## Supplementary Material

gkac027_Supplemental_Files
