# Supplementary material for: Waves of sumoylation support transcription dynamics during adipocyte differentiation
Source: Nucleic Acids Res. 2022 Jan 31;50(3):1351–69. doi: 10.1093/nar/gkac027 (PMC8860575; doi:10.1093/nar/gkac027)
Supplement: gkac027_Supplemental_Files [file gkac027_supplemental_files.zip › Supplementary Material_revised_R2.pdf]

Supplementary figures and legends

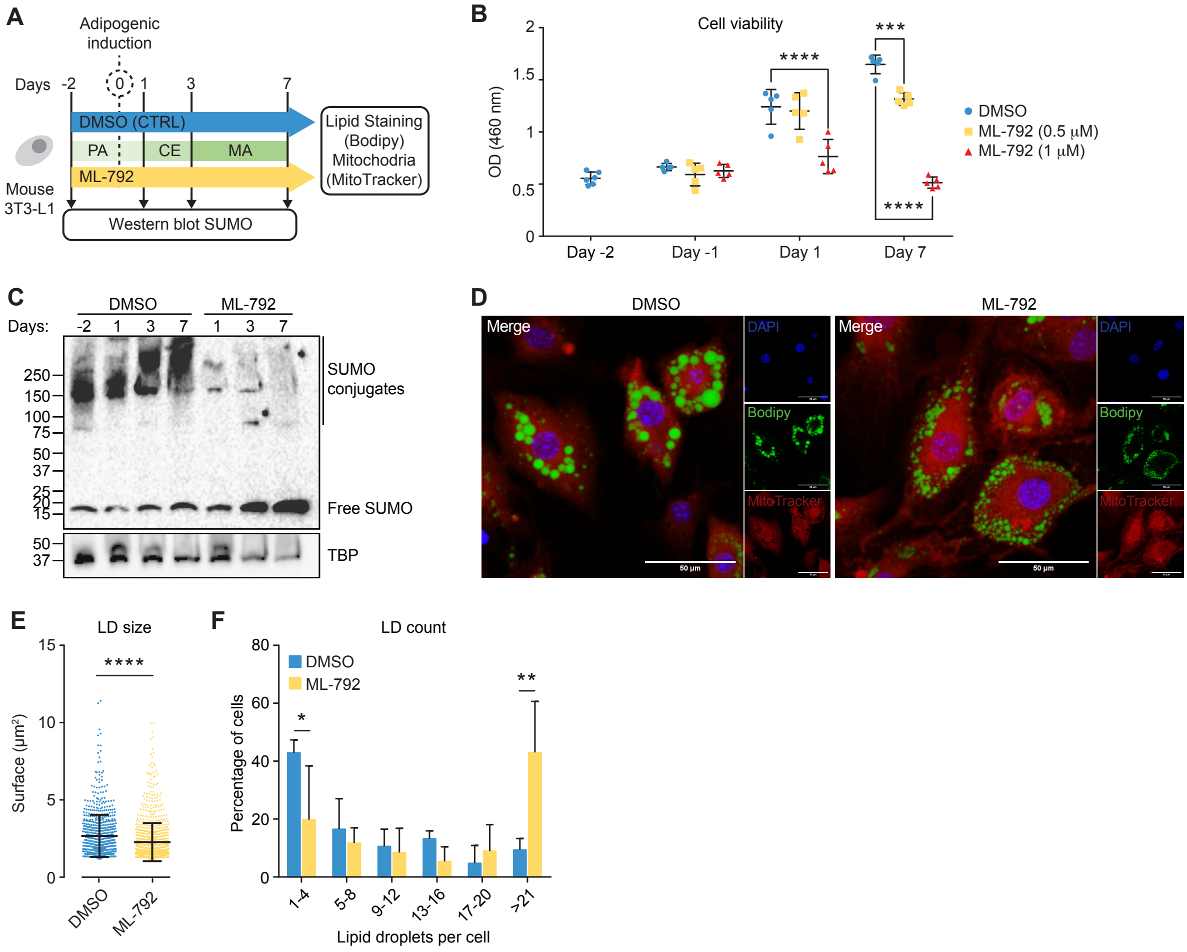

Supplementary Figure S1. Sumoylation inhibition triggers lipoatrophy

(A) Experimental layout of Western blot and lipid droplets staining time-course experiments.

(B) Effect of 0.5 µM and 1 µM of ML-792 on 3T3L1 cells viability during adipogenesis.

(C) Western blot analysis of SUMO-2/3 during adipogenesis in DMSO and ML-792-treated 3T3-L1 cells. Cells treated with DMSO or ML-792 (0.5µM) were collected at indicated time points. Cell lysates were subjected to Western blot using a SUMO-2/3 antibody. TBP was used as loading control.

(D) Immunofluorescence analysis of DMSO or ML-792 treated adipocytes (Day 7). Lipid droplets were stained with the lipid probe Bodipy. Nuclei were counterstained with DAPI. To facilitate lipid droplet quantification mitochondria were stained with MitoTracker. Images were acquired using a Leica confocal SP8 device (40x). Images are representative of at least 3 biological replicates.

(E) Effect of ML-792 on the size of lipid droplets. The size of lipid droplets at Day 7 was measured using the FIJI software. The surface area is measured in µm^2 (y axis). At least 4 representative fields

per well were analyzed. The significance of mean comparison is determined by unpaired t-test. ****: $p \leq 0.0001$.

(F) Effect of ML-792 on the number of lipid droplets per cell. The graph displays the percentage of adipocytes (y axis) in subpopulations based on lipid droplet number per cell (x axis). Results are representative of 3 biological replicates. Statistical analysis was performed using two-way ANOVA and Sidak's multiple comparisons tests. *: $p \leq 0.05$ and **: $p \leq 0.01$.

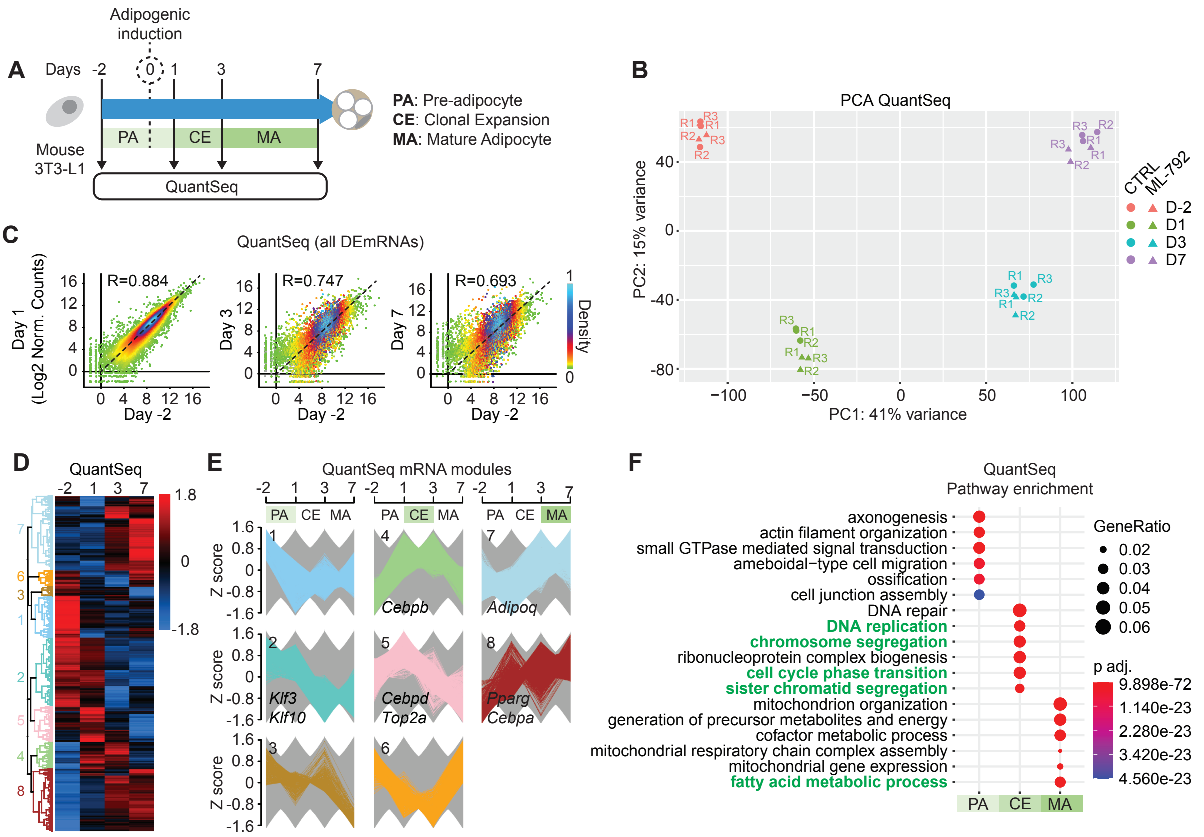

Supplementary Figure S2. Characterization of total mRNA dynamics during adipogenesis using QuantSeq.

(A) Experimental layout of QuantSeq time-course experiments

(B) PCA of QuantSeq data.

(C) Scatter plots showing total mRNA levels for all QuantSeq time-course differentially expressed mRNAs at each time point versus day −2. Differentially expressed mRNAs were identified based on statistical differences between conditions using a p-adj cutoff < 0.01 and mRNA levels are presented as log2 transformed normalized counts. R, Pearson correlation coefficient.

(D, E) Hierarchical clustering of QuantSeq differentially expressed mRNAs. Clusters *(D)* are categorized in PA- CE and MA-specific modules *(E)*: PA, pre-adipocyte; CE, clonal expansion; MA, mature adipocyte. Z score is calculated for log2 transformed normalized counts. Module numbers *(E)* are indicated on the left side of the heatmap in *(D)*.

(F) Pathway enrichment analysis of QuantSeq mRNA modules. PA, pre-adipocyte; CE, clonal expansion; MA, mature adipocyte.

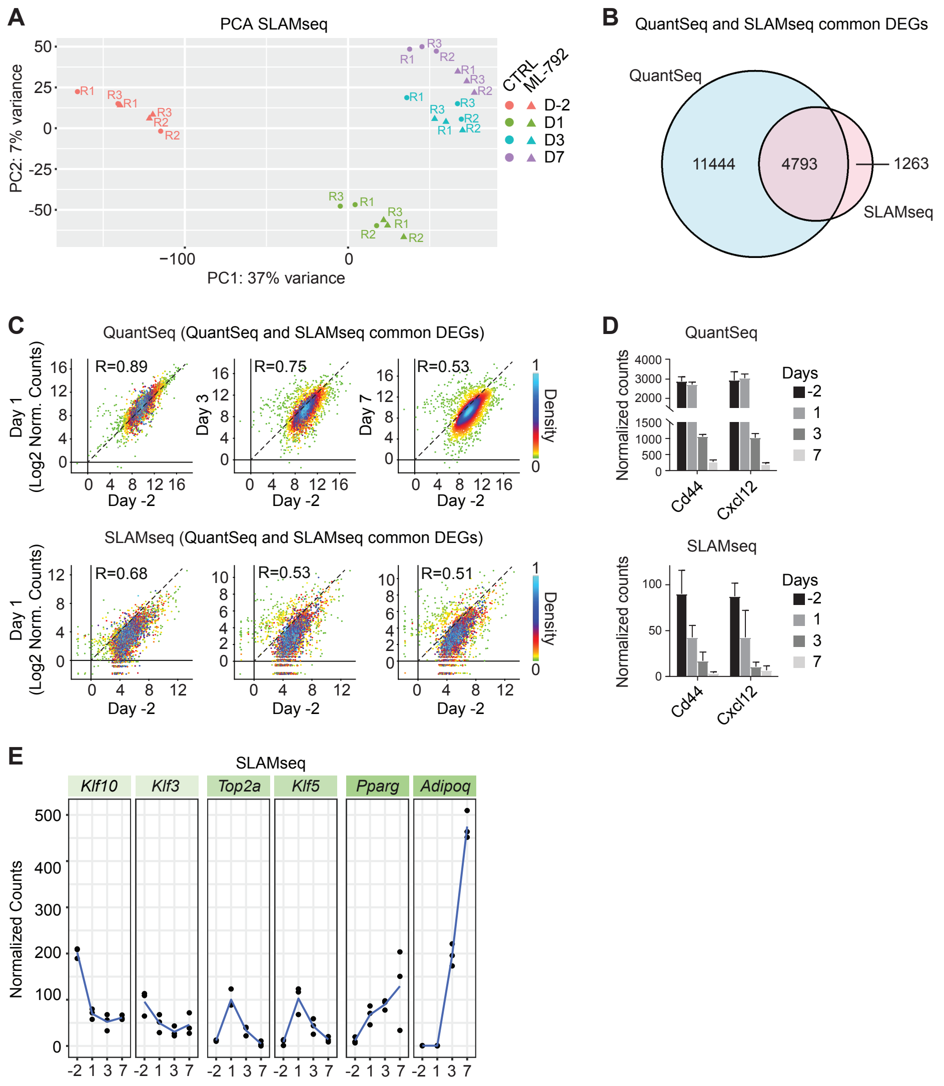

Supplementary Figure S3. Comparision between QuantSeq and SLAMseq results.

(A) PCA of SLAMseq data.

(B) Venn diagram featuring QuantSeq and SLAMseq common DEGs.

(C) Scatter plots showing expression profiles of common QuantSeq and SLAMseq DEGs. DEGs were identified based on statistical differences between conditions using a p-adj cutoff < 0.01. Upper panel: QuantSeq data for common DEGs; Lower panel: SLAMseq data for common DEGs. The nascent (SLAMseq) and total mRNA (QuantSeq) levels are presented as log2 transformed normalized counts. R, Pearson correlation coefficient.

(D) Examples of genes showing differences expression dynamics between QuantSeq and SLAMseq.

(E) Profiles of representative transcripts in SLAMseq time-course modules. The y axis is presented as normalized read counts.

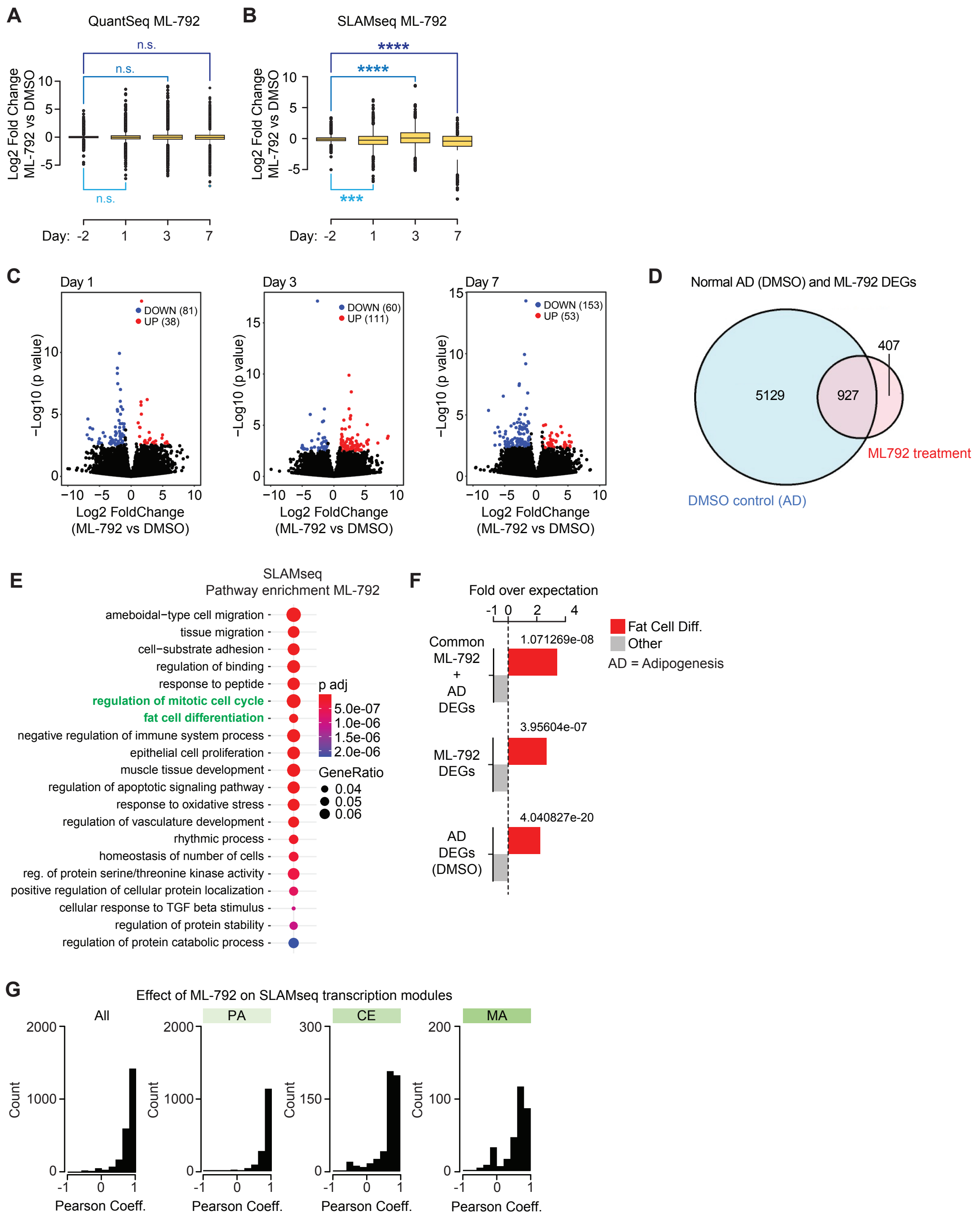

Supplementary Figure S4. ML-792 affects transcription more significantly than total mRNA levels.

(A) Effects of ML-792 (log2 fold change ML-792 vs DMSO) on total mRNA levels across time points in Quant-seq. Statistical significance was assessed using one-way ANOVA followed by multiple comparisons test. Non-significant: $p > 0.05$

(B) Effects of ML-792 (log2 fold change ML-792 vs DMSO) on nascent transcripts across time points in SLAM-seq. Statistical significance was assessed using one-way ANOVA followed by multiple comparisons test. ***: $p \leq 0.001$, ****: $p \leq 0.0001$.

(C) Effects of ML-792 (log2 fold change ML-792 vs DMSO) on nascent transcripts at each time point in SLAM-seq. Volcano plots were generated after DEGs were determine using Wald test. Colored dots show downregulated (blue) and upregulated (red) DEGs with log2 fold change > 2 and p-adj < 0.1. The number of DEGs in each group is shown between brackets.

(D)Venn diagram featuring DMSO control and ML-792 time-course SLAMseq DEGs.

(E) Pathway enrichment analysis of the genes regulated in presence of ML-792 (SLAMseq DEGs).

(F) Enrichment of DEGs belonging to the fat cell differentiation GO term in subsets of DEGs defined in *(C)*. Hypergeometric test was used for testing significance.

(G) Distribution of Pearson correlation coefficients in transcription modules. The y axis displays transcript count numbers. The x axis displays Pearson correlation coefficients and is segmented into 10 bins. PA, pre-adipocyte; CE, clonal expansion; MA, mature adipocyte.

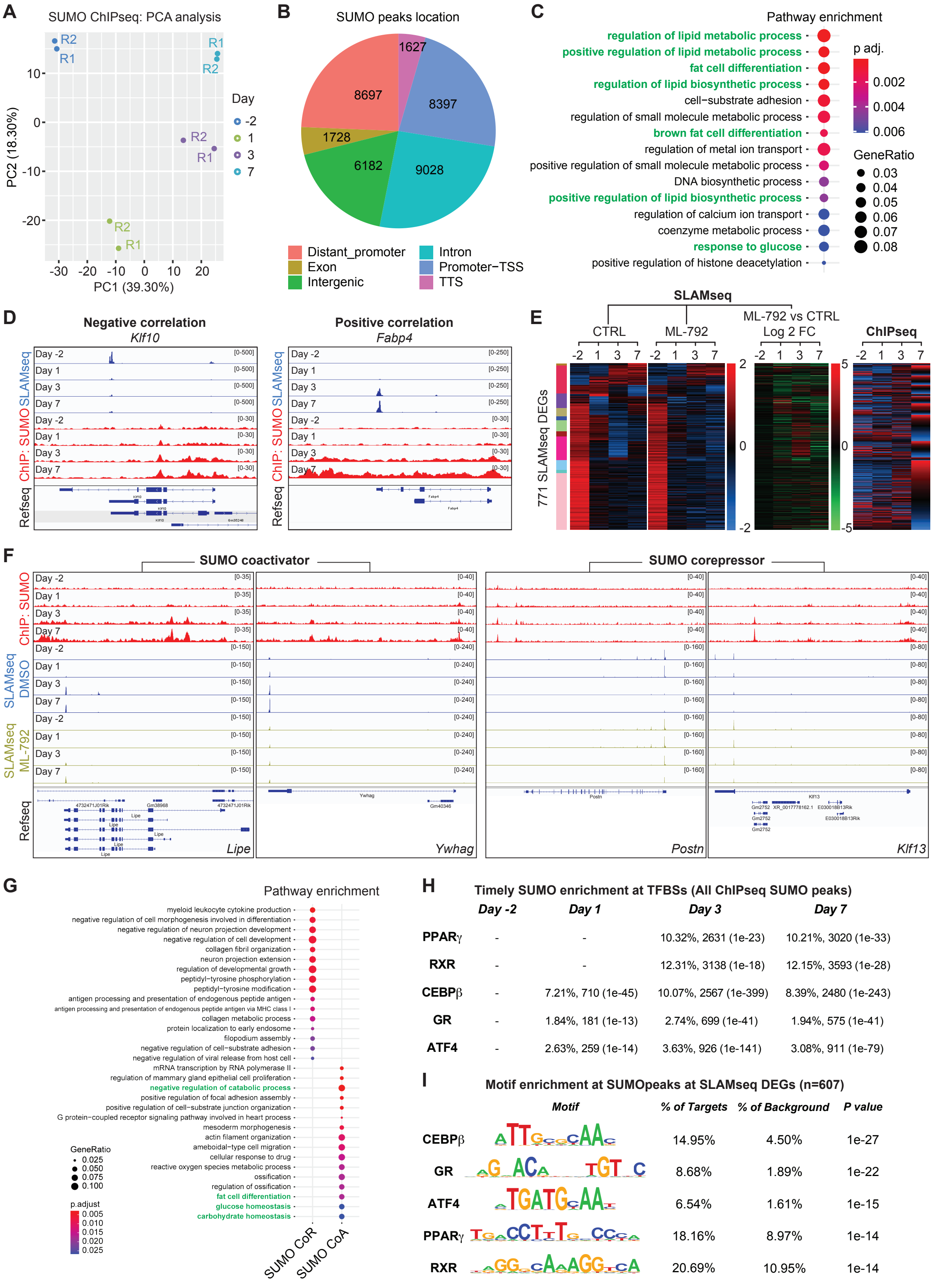

Supplementary Figure S5. Characterisation of the SUMO chromatin landscape of differentiating 3T3-L1 cells

(A) PCA of SUMO ChIPseq biological replicates.

(B) Genome-wide distribution of SUMO peaks.

(C) Pathway enrichment analysis of all genes showing a significantly dynamic binding of SUMO during adipogenesis.

(D) Snapshot from the IGV genome browser for ChIPseq and SLAMseq data at *Klf10* (negative correlation) and *Fabp4* (positive correlation) loci.

(E) Heatmaps presenting the comparison of SLAMseq in CTRL (DMSO, *see Figure. 1D)*, SLAMseq in ML-792 and SUMO ChIPseq during adipogenesis. Log2 fold change between ML-792 and DMSO treatments were calculated.

(F) Snapshot from the IGV genome browser for ChIPseq and SLAMseq data at two genes representative of the role of SUMO as coactivator (Left: *Lipe* and *Ywhag*) and two genes representative of the role of SUMO as corepressor (Right: *Postn* and *Klf13*).

(G) Pathway enrichment analysis of genes at which SUMO acts as corepressor (CoR) or coactivator (CoA).

(H) Snapshot of the unbiased analysis of TFBS enrichment at SUMO peaks as depicted in Figure. 3L. (Complete list in Supplementary Table S6).

(I) Enrichment of TFBSs at SUMO peaks found at SLAMseq DEGs.

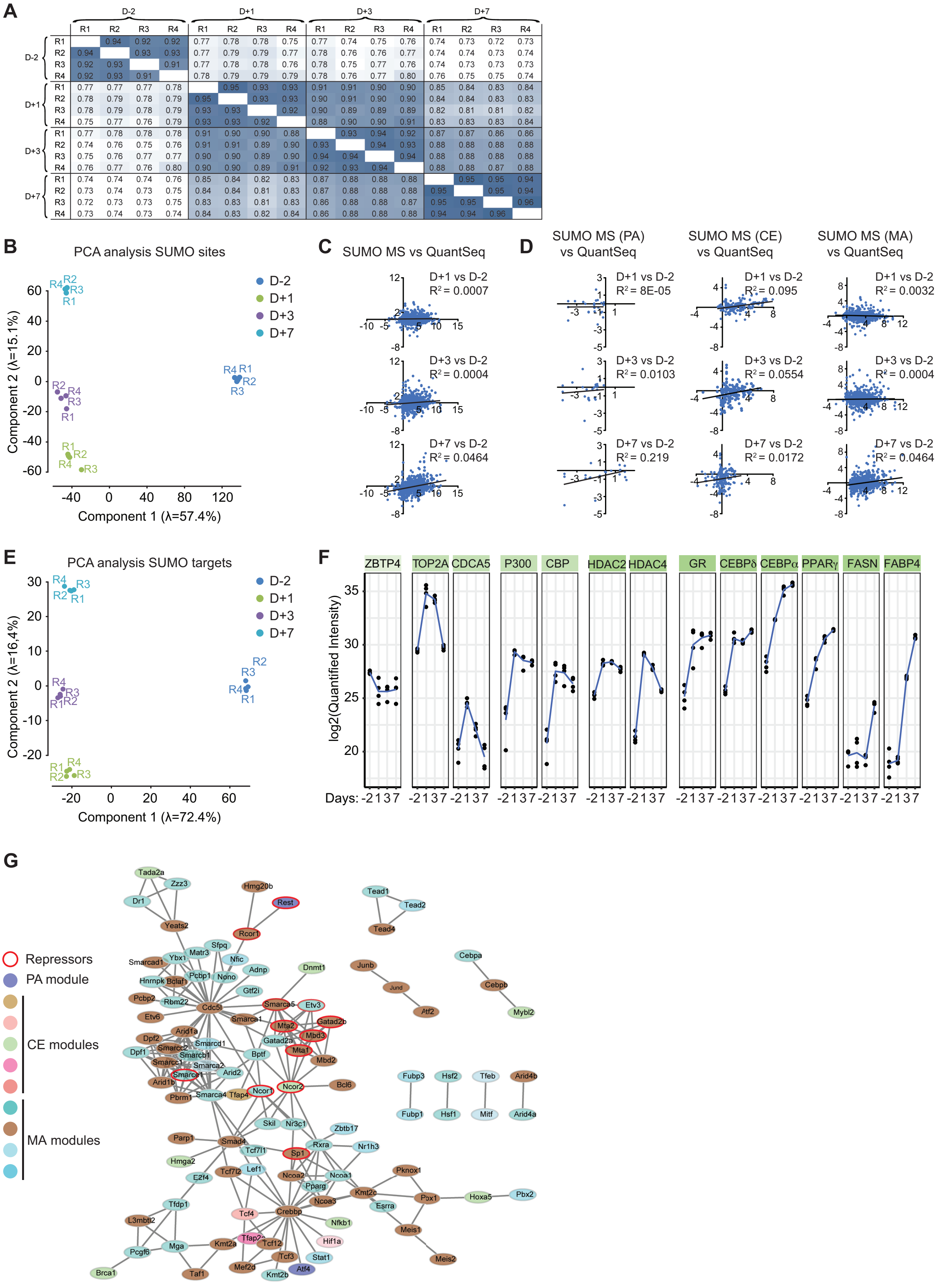

Supplementary Figure S6. Site specific characterization of the endogenous SUMOylome of differentiating adipocytes.

(A) Pearson correlation analysis of MS biological replicates (n = 4).

(B) PCA of site-specific sumoylation across samples.

(C) Correlation between whole transcriptome data (QuantSeq) and SUMO targets (SUMO MS) at each time point during adipogenesis. x axis: log2 fold change (SUMO MS); y axis: log2 fold change (Quantseq).

(D) Correlation between whole transcriptome data (QuantSeq) and SUMO targets (SUMO MS) in each sumoylation module. PA, pre-adipocyte; CE, clonal expansion; MA, mature adipocyte. x axis: log2 fold change (SUMO MS); y axis: log2 fold change (Quantseq).

(E) Pearson correlation analysis of SUMO targets.

(F) Sumoylation profiles of representative SUMO targets in sumoylation modules.

(G) STRING network analysis of sumoylated TFs. Node color: same as the colors of sumoylation module; Red outlines indicate transcriptional repressor.

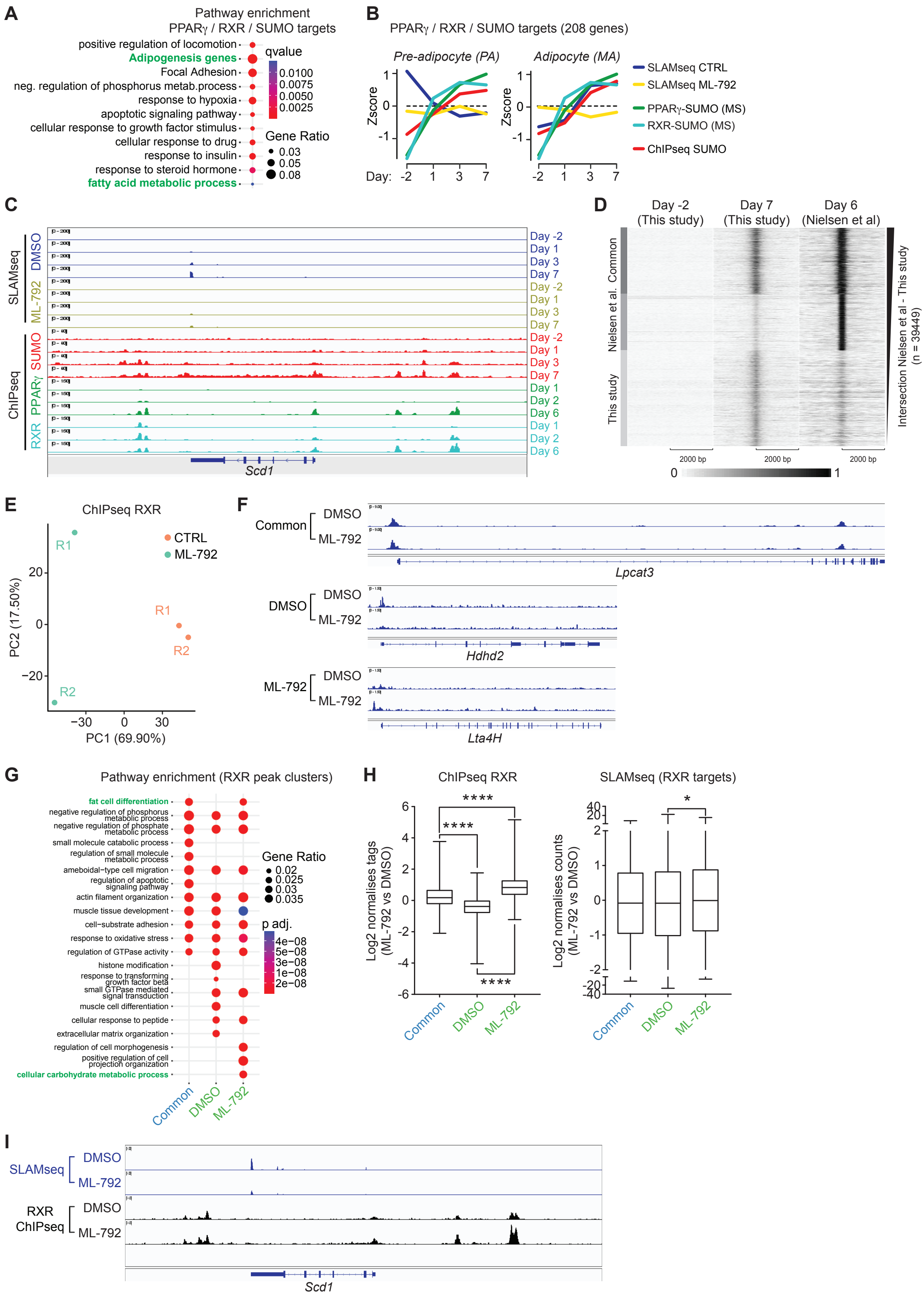

Supplementary Figure S7. Effect of ML-792 on the PPARγ, RXR and SUMO regulon.

(A) Pathway enrichment analysis of PPARγ, RXR and SUMO common targets.

(B) Loess regression plots showing transcription profiles (CTRL, blue; ML-792, yellow), SUMO binding at PPRE (red) and SUMOylated PPARγ (green) and RXR (Turquoise) at PPARγ, RXR and SUMO common target genes. Z scores are calculated for log2 transformed normalized counts (transcript or SUMO binding site) or log2 transformed label-free quantified (LFQ) values (SUMO proteomics).

(C) Snapshot of the IGV genome browser showing the activation of the PPARγ/RXR target gene *Scd1* during AD (SLAMseq, DMSO), the downregulation of *Scd1* after ML-792 treatment (SLAMseq, ML-792) and the coordinated recruitment of SUMO, PPARγ and RXR at *Scd1* during AD (ChIPseq). PPARγ and RXR ChIPseq data originate from Nielsen et al (1).

(D) Comparison of RXR ChIPseq experiments performed in this study and in Nielsen et al (1). Peaks were sorted according to levels of intersection between studies and conditions.

(E) PCA of our RXR ChIPseq experiment across samples.

(F) Snapshot of the IGV genome browser showing representative examples genes belonging to the RXR ChIPseq clusters (“Common”, “DMSO” and “ML-792”) described in *Figure 5E*.

(G) Pathway enrichment analysis of RXR target genes belonging to the three clusters described in *Figure 5E*.

(H) Comparison of the effect of ML-792 on RXR presence at the chromnatin (ChIPseq) and RXR target genes transcription levels (SLAMseq). The results are represented as log2 normalized counts for SLAMseq and Log2 normalized tags for ChIPseq (ML-792 versus DMSO). The significance of mean comparison was determine using one-way ANOVA followed by multiple comparisons test. *: $p \leq 0.05$, ****: $p \leq 0.0001$.

(I) Snapshot of the IGV genome browser showing the down-regulation of the MA gene *Scd1* (SLAMseq) and the concomitant increased of the presence of RXR at the chromatin (ChIPseq).

Supplementary table list and legends

Supplementary Table S1: Characterization of total mRNA dynamics using QuantSeq

Supplementary Table S2: Characterization of transcription dynamics using SLAMseq

Supplementary Table S3: Effect of ML-792 on total mRNA dynamics (ML792vsDMSO)

Supplementary Table S4: Effect of ML-792 on transcription dynamics (ML792vsDMSO)

Supplementary Table S5: Characterization of the SUMO-chromatin landscape dynamics using ChIPseq

Supplementary Table S6: Unbiased and targeted motif enrichment at SUMO ChIPseq peaks

Supplementary Table S7: Integration of PPARγ, RXR and SUMO ChIPseq datasets

Supplementary Table S8: Identification of endogenous sumoylation sites by mass spectrometry

Supplementary Table S9: characterization of PPARγ, RXR and SUMO common targets by ChIPseq

Supplementary Table S10: Characterization of RXR peaks in DMSO and ML-792 by ChIPseq

1. Nielsen, R., Pedersen, T.A., Hagenbeek, D., Moulos, P., Siersbaek, R., Megens, E., Denissov, S., Borgesen, M., Francoijs, K.J., Mandrup, S. *et al.* (2008) Genome-wide profiling of PPARgamma:RXR and RNA polymerase II occupancy reveals temporal activation of distinct metabolic pathways and changes in RXR dimer composition during adipogenesis. *Genes Dev*, **22**, 2953-2967.
